# Dissection of transcriptional events in graft incompatible reactions of “Bearss” lemon (*Citrus limon*) and “Valencia” sweet orange (*C. sinensis*) on a novel citrandarin (*C. reticulata × Poncirus trifoliata*) rootstock

**DOI:** 10.3389/fpls.2024.1421734

**Published:** 2024-06-20

**Authors:** Vicente J. Febres, Anas Fadli, Bo Meyering, Fahong Yu, Kim D. Bowman, Jose Xavier Chaparro, Ute Albrecht

**Affiliations:** ^1^ Horticultural Sciences Department, University of Florida/Institute of Food and Agricultural Sciences (IFAS), Gainesville, FL, United States; ^2^ Southwest Florida Research and Education Center, University of Florida/Institute of Food and Agricultural Sciences (IFAS), Immokalee, FL, United States; ^3^ Interdisciplinary Center for Biotechnology Research (ICBR), University of Florida, Gainesville, FL, United States; ^4^ Horticultural Research Laboratory, United States Department of Agriculture (USDA), Fort Pierce, FL, United States

**Keywords:** citrus, graft compatibility, graft incompatibility, RNA-seq, differentially expressed genes, stress response, plant defense, rootstock

## Abstract

Citrus is commercially propagated via grafting, which ensures trees have consistent fruit traits combined with favorable traits from the rootstock such as soil adaptability, vigor, and resistance to soil pathogens. Graft incompatibility can occur when the scion and rootstock are not able to form a permanent, healthy union. Understanding and preventing graft incompatibility is of great importance in the breeding of new fruit cultivars and in the choice of scion and rootstock by growers. The rootstock US-1283, a citrandarin generated from a cross of “Ninkat” mandarin (*Citrus reticulata*) and “Gotha Road” #6 trifoliate orange (*Poncirus trifoliata*), was released after years of field evaluation because of its superior productivity and good fruit quality on “Hamlin” sweet orange (*C. sinensis*) under Florida’s growing conditions. Subsequently, it was observed that trees of “Bearss” lemon (*C. limon*) and “Valencia” sweet orange (*C. sinensis*) grafted onto US-1283 exhibited unhealthy growth near the graft union. The incompatibility manifested as stem grooving and necrosis underneath the bark on the rootstock side of the graft. Another citrandarin rootstock, US-812 (*C. reticulata* “Sunki” × *P. trifoliata* “Benecke”), is fully graft compatible with the same scions. Transcriptome analysis was performed on the vascular tissues above and below the graft union of US-812 and US-1283 graft combinations with “Bearss” and “Valencia” to identify expression networks associated with incompatibility and help understand the processes and potential causes of incompatibility. Transcriptional reprogramming was stronger in the incompatible rootstock than in the grafted scions. Differentially expressed genes (DEGs) in US-1283, but not the scions, were associated with oxidative stress and plant defense, among others, similar to a pathogen-induced immune response localized to the rootstock; however, no pathogen infection was detected. Therefore, it is hypothesized that this response could have been triggered by signaling miscommunications between rootstock and scion either through (1) unknown molecules from the scion that were perceived as danger signals by the rootstock, (2) missing signals from the scion or missing receptors in the rootstock necessary for the formation of a healthy graft union, (3) the overall perception of the scion by the rootstock as non-self, or (4) a combination of the above.

## 1 Introduction

Commercial production of citrus fruits relies on grafting for the vegetative propagation of cultivars. This guarantees that the fruiting variety is true-to-type, has a short juvenility period and improves soil adaptability and tolerance or resistance to soil pathogens and associated stresses (Bowman and Joubert, 2020). Additionally, rootstocks can modulate and improve certain characteristics to the scion, such as vigor and fruit quality (Wheaton et al., 1995; Bowman and Joubert, 2020; Morales Alfaro et al., 2023). Therefore, the establishment, and long-term survival and health of the graft union is crucial. The formation of functional graft unions involves complex processes that include tissue adhesion, followed by the production of callus between the grafting partners, the development of new cambium, and the connection of vascular tissues between the two parts to form a single functional unit (Melnyk et al., 2015; Rasool et al., 2020). Graft unions that result in healthy growth of the scion–rootstock combination are considered compatible. In those combinations where the rootstock and scion fail to establish a strong and lasting connection, graft incompatibility ensues. This occurrence, which manifests as anatomical abnormalities at the union, stunted growth, or even complete failure of the graft and death of the plant can take months or years to develop (Bevington, 1976; Melnyk, 2017; de Carvalho et al., 2018).

Results in Arabidopsis indicate that in the initial stages of graft union formation, prior to contact between the two parts, differential gene expression, cell division, and cell expansion between rootstock and scion occur (Melnyk et al., 2015). This asymmetry lessens once contact between the grafting partners is established. The genetic requirements for the subsequent vascular reconnection between scion and rootstock are also different, with two auxin-responsive genes being important below, but not above, the graft junction (Melnyk et al., 2015). This is suggestive of recognition by the rootstock of a signal from the scion for the successful formation of graft unions (Melnyk et al., 2015; Tedesco et al., 2022). Conversely, a breakdown in signaling between the two parts could lead to the recognition of the other partner in the graft as non-self, leading to incompatibility (Tanaka and Heil, 2021; Ge et al., 2022; Tedesco et al., 2022).

Graft incompatibility can be classified into (1) translocated incompatibility, which manifests as leaf chlorosis, retardation or cessation of scion growth, and/or poor development of the root system within the first year after grafting; (2) localized incompatibility, in which a disruption of the vascular continuity between rootstock and scion occurs and may take years to manifest; and (3) virus-induced incompatibility, in which viral infections result in the disruption of an otherwise compatible graft union (Rasool et al., 2020). For instance, certain strains of *Citrus tristeza virus* (CTV), can cause the decline of various citrus scions grafted onto sour orange rootstock as a result of cell death at the graft union (Moreno et al., 2008).

The formation of the graft union is genetically controlled and associated with the transcriptional reprogramming of biological processes such as wound response, cell wall modifications, cell division and differentiation, and hormonal signaling (Cookson et al., 2013; Melnyk et al., 2015; Pina et al., 2017; Habibi et al., 2022). These processes can be sensitive to the genetic distance between the scion and the rootstock. The greater the genetic divergence between the grafting partners, the more complex the interactions become, increasing the likelihood of graft failure (Gautier et al., 2019). Additionally, as in the case of citrus, the compatibility between the grafting partners can also be influenced by their respective position relative to the graft union (Raiol-Junior et al., 2022). Certain genotypes exhibit higher compatibility when used as scions, while others demonstrate greater compatibility when used as rootstocks or as interstocks.

US-1283 (*Citrus reticulata* “Ninkat” × *Poncirus trifoliata* “Gotha Road” #6) is a hybrid citrus rootstock that was released in 2014 after more than 14 years of field evaluations by the U.S. Department of Agriculture (USDA) (Bowman and McCollum, 2014). These evaluations were conducted using “Hamlin” sweet orange as the scion. During subsequent propagations for new field trials with additional scions, including “Bearss” lemon, “Star Ruby” grapefruit, and “Tango” mandarin, abnormal growth that appeared to be graft incompatibility was observed in these three combinations (Bowman and Albrecht, 2020). The rootstock US-812 (*C. reticulata* “Sunki × *P. trifoliata* “Benecke”), another mandarin × trifoliate orange hybrid rootstock, released by the USDA in 2005 (Bowman and Rouse, 2006), does not show incompatibility with any of those scions. Therefore, we sought out to compare the graft responses of these two rootstocks. The main objective of this study was to identify pathways, expression networks, and molecular mechanisms associated with incompatibility by analyzing transcriptome changes in rootstocks with similar genetic backgrounds but different responses to grafting. We reasoned that performing a transcriptome analysis of the vascular tissues near the graft union of compatible and incompatible graft combinations would help understand the processes and potential causes of incompatibility in citrus. Graft compatibility in citrus has not been studied extensively. Presently, compatibility can only be assessed by the grafting of each new rootstock and scion combination, followed by multi-year field evaluations. Given the increased numbers of novel scions and rootstocks developed by citrus breeders and the importance of grafting for propagation, there is an urgent need to develop effective and fast tools to understand and diagnose graft incompatibility and prevent consequent unexpected tree loss in the nursery and the field. This study is also an initial step in the identification of potential gene expression targets for marker development.

## 2 Materials and methods

### 2.1 Plant materials

Two rootstocks were used in this study, US-1283 [“Ninkat” mandarin (*C. reticulata*) × “Gotha Road” #6 trifoliate orange (*P. trifoliata*)] and US-812 [“Sunki” mandarin (*C. reticulata*) × “Benecke” trifoliate orange (*P. trifoliata*)]. Seeds from both rootstocks were sown into 1.5” × 8.25” (3.8 cm × 21 cm) Ray Leach cone-tainers (Stuewe & Sons, Tangent, OR) containing Fafard 4P potting medium (Fafard, Agawam, MA) and transplanted into 5” × 9.5” (12.7 cm × 24.1 cm) tree pots (Stuewe & Sons) containing the same potting medium after 9 months. Rootstock liners were grafted by the inverted T-bud method (Albrecht et al., 2021) with certified, disease-free budwood of “Valencia” sweet orange (*C. sinensis*) and “Bearss” lemon (*C. limon*). Buds were grafted 10 cm above the soil level. A total of 6 to 15 plants per graft combination were produced. Buds were unwrapped after 2–3 weeks when the unions had healed and produced callus. At this point, rootstock liners were broken above the graft union and bent to force shoot growth. Rootstocks were cut off when scions had grown by at least 10 cm. Plants were grown in an enclosed temperature-controlled (13–33°C) greenhouse under natural light at the University of Florida, IFAS Southwest Florida Research and Education Center, Immokalee, FL.

### 2.2 RNA extraction and Illumina sequencing library construction

Stem vascular tissues were collected for RNA extraction 12 months after grafting from both compatible and incompatible combinations after the full expression of graft incompatibility symptoms had developed (fluting or grooving of the rootstock trunk; see Results section). The outer layer of bark was first removed and, using a potato peeler, thin strips of phloem and xylem tissue were collected from 2 cm above and below the graft union and immediately frozen in liquid nitrogen. The tissue was ground in liquid nitrogen using mortar and pestle and stored at −80°C until use. Approximately 100 mg of ground tissue was used to extract RNA using the Qiagen RNeasy plant mini kit (Qiagen, Valencia, CA) according to the manufacturer’s instructions. RNA quality and concentration were measured using the QUBIT RNA fluorometry method (Invitrogen, Waltham, MA) and an Agilent Bioanalyzer (Agilent, Santa Clara, CA). An amount of 250 ng of high-quality total RNA with an RNA integrity number (RIN) of 7 or higher was used for the library construction using the reagents provided in the NEBNext Poly(A) mRNA Magnetic Isolation Module (New England Biolabs, Ipswich, MA) and the NEBNext Ultra II Directional RNA Library Prep Kit (New England Biolabs) according to the manufacturer’s instructions. Briefly, 100 ng of total RNA was used for mRNA isolation using the NEBNext Poly(A) mRNA Magnetic Isolation Module (New England Biolabs). The poly A enriched RNA was fragmented in NEBNext First Strand Synthesis Buffer via incubation at 94°C for 8 min. This step was followed by first-strand cDNA synthesis using reverse transcriptase and random hexamer primer. Synthesis of double-stranded cDNA was done using the second-strand master mix provided in the kit, followed by end-repair and dA-tailing. At this point, Illumina adaptors were ligated to the sample. Finally, the library was amplified, followed by purification with AMPure beads (Beckman Coulter, Pasadena, CA). The library size and mass were assessed by analysis in the Agilent TapeStation using a High Sensitivity DNA1000 Screen Tape. Typically, a 200–1,000 broad library peak is observed with the highest peak at ~500 bp. Thirty-nine barcoded libraries were pooled equimolarly for sequencing simultaneously for 0.52 lanes of NavaSeq 6000 S4 2 × 150 cycles run as described below. RNA-Seq library was performed at the UF ICBR Gene Expression Core (https://biotech.ufl.edu/gene-expression-genotyping/, RRID: SCR_019145).

### 2.3 Illumina NovaSeq6000 sequencing

Normalized libraries were submitted to the Illumina “Free Adapter Blocking Reagent” protocol (Illumina, San Diego, CA) to minimize the presence of adaptor-dimers and index hopping rates. The library pool was diluted to 0.8 nM and sequenced on one S4 flow cell lane (2 × 150 cycles) of the Illumina NovaSeq6000. The instrument’s computer utilized the NovaSeq Control Software v1.6. Cluster and SBS consumables were v1.5. The final loading concentration of the library was 120 pM with 1% PhiX spike-in control. One lane generated 2.5–3 billion paired-end reads (~950 Gb) with an average Q30% ≥92.5% and Cluster PF = 85.4%. FastQ files were generated using the BCL2fastQ function in the Illumina BaseSpace portal. The Illumina NovaSeq 6000 was used to sequence the libraries for 2 × 150 cycles. Sequencing was performed at the ICBR NextGen Sequencing (https://biotech.ufl.edu/next-gen-dna/, RRID: SCR_019152).

### 2.4 Annotation and expression analysis

Reads acquired from the Illumina NovaSeq 6000 platform were cleaned up with the cutadapt program (Martin, 2011) to trim off sequencing adaptors and low-quality bases with a quality phred-like score <20. Reads <40 bases were excluded from RNA-seq analysis. The *Citrus clementia* (v1.0_182) genome from JGI (Joint Genome Institute, Berkeley, CA) was used as reference sequence for RNA-seq analysis. The cleaned reads of each sample were mapped to the reference genome using the read mapper of the STAR package (Spliced Transcripts Alignment to a Reference, v2.7.9a) (Dobin et al., 2012). The mapping results were processed with the HTSeq (High-Throughput Sequence Analysis in Python, v0.11.2) (Anders et al., 2014), samtools, and scripts developed in-house at ICBR of UF to remove potential PCR duplicates, and choose and count uniquely mapped reads for gene expression analysis. Principal component analysis (for detecting outlier samples) based on all identified genes in each analysis was performed with the R-package v4.1.3 (R Core Team, 2022). The counted reads of each gene were analyzed by an in-house DESeq2-based R pipeline. The adjusted *p*-value was used to detect the false-positive rate in each comparison. Significant up- and downregulated genes were selected using the *p*-value, log_2_ fold change (log2FC), and the adjusted *p*-value (*p*_Adj_) for downstream analysis. The RNA-Seq data reported here have been deposited in NCBI’s Gene Expression Omnibus (GEO), accession number GSE263656.

### 2.5 Gene enrichment analysis

Gene ontology (GO) and Kyoto Encyclopedia of Genes and Genomes (KEGG) pathway enrichment analysis was performed using the g:GOSt version e109_eg56_p17_1d3191d online tool in the g:Profiler public web server (Kolberg et al., 2023) with *C. clementina* selected as the organism, a Set Counts and Sizes algorithm (g:SCS) significance threshold of 0.05, and the “highlight driver terms in GO” option selected. Only differentially expressed genes (DEGs) with *p*_Adj_ < 0.05 were included. Within these, genes with a log2FC ≥1 were considered as induced, and genes with a log2FC ≤−1 were considered as repressed. g:GOSt generated a list of enriched terms, and from the statistically significant enriched terms, those deemed non-redundant based on the topology of the annotation (terms that are not directly connected) and with the smallest adjusted *p*-value (*p*_Adj_) were highlighted. The KEGG mapper reconstruction tool (Kanehisa et al., 2023) was used to map KEGG pathway and other networks using the annotated K numbers (KO identifiers). JMP Genomics v9.1 was used to generate bubble plots and heatmaps to represent the results in graphical form.

### 2.6 Quantitative real-time PCR analysis

Twelve genes involved in different metabolic pathways were selected for qPCR validation (**Supplementary Table S1**). Specific primers were designed based on the cDNA sequence available on the Joint Genome Institute (JGI) using the IDT PrimerQuest™ tool. BLAST and MEGA 11.0.13 (Tamura et al., 2021) were used to verify the sequence specificity of the designed primers. Total RNA (1 µg) was used for cDNA synthesis using the iScriptTM gDNA Clear cDNA Synthesis Kit (Bio-Rad, Hercules, CA) following the manufacturer’s instructions. The primer pairs were tested for qRT-PCR specificity using melting-curve analysis. qRT-PCR was performed using the SsoAdvanced Universal SYBR^®^ Green Supermix (Bio-Rad) on a CFX96 qPCR System (Bio-Rad) in a total volume of 20 µL, which consisted of 10 µL of SYBR^®^ Green Supermix, 1 µL of each specific primer (equivalent to 500 nM per reaction), 2 µL of 1:10 diluted cDNA (equivalent to 10 ng per reaction), and 6 µL of nuclease-free water. Thermal cycling conditions were 30 s of denaturation at 95°C followed by 40 cycles of 95°C for 15 s and annealing at 60°C for 30 s. All reactions were performed in triplicate with three biological replicates. The tubulin gene was used as an endogenous control gene (**Supplementary Table S1**). Relative gene expression was calculated using the 2^−ΔΔCt^ method (Livak and Schmittgen, 2001).

## 3 Results

We set out to compare compatible and incompatible graft reactions on US-1283 rootstock. Prior observations during nursery propagations indicated that “Bearss” lemon grafted onto US-1283 exhibited abnormal graft unions within a few months after grafting, while “Valencia” sweet orange did not. In our follow-up grafting study, however, “Valencia” developed abnormalities similar to “Bearss” in combination with US-1283, although the symptoms appeared several weeks later and were less severe. Therefore, for the subsequent transcriptome analysis, we focused on pairwise comparisons between both incompatible reactions on US-1283 and the respective compatible ones on US-812.

### 3.1 Morphological and anatomical response

**Figure 1:**
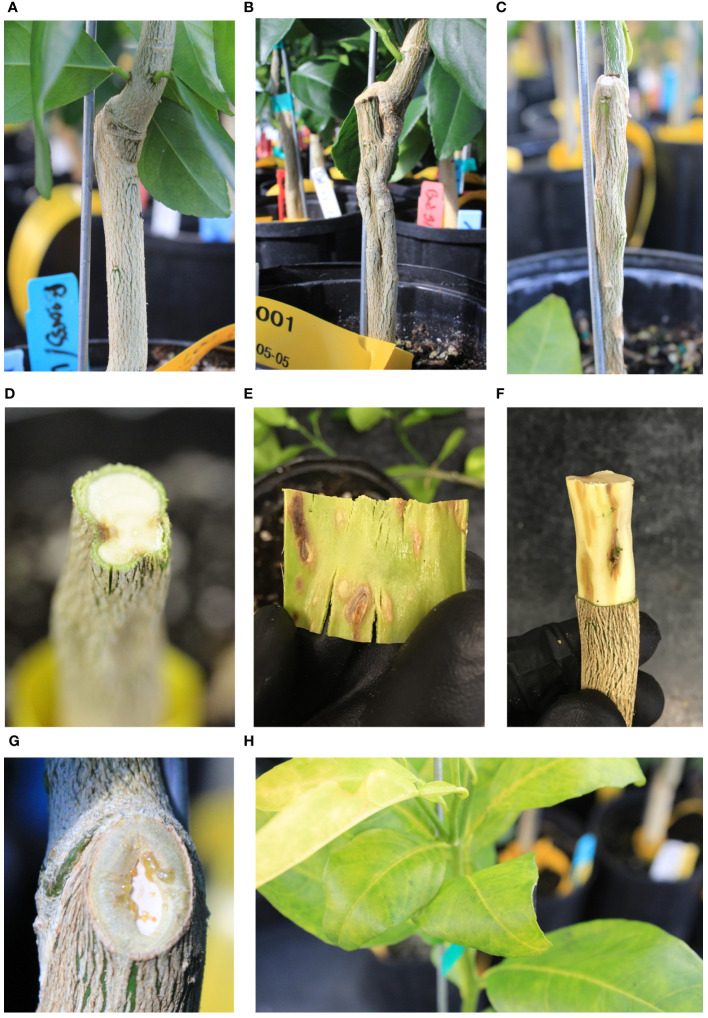
Grafted plants morphology. **(A)** US-812 grafted with “Bearss” lemon (BL/US-812, compatible). **(B)** Grooving in the rootstock and scion swelling above the graft union in BL/US-1283 (incompatible). **(C)** Grooving in the US-1283 rootstock grafted with “Valencia” sweet orange (VL/US-1283, incompatible). **(D)** Cross section of the rootstock stem of BL/US-1283 (incompatible) showing necrosis and grooving. **(E)** Necrosis in the rootstock bark (inner side shown) of BL/US-1283 (incompatible). **(F)** Pitting and necrosis in the wood of US-1283 grafted with “Bearss” lemon (BL/US-1283, incompatible). **(G)** Sap exudate in BL/US-1283 (incompatible) near the graft union. **(H)** Leaf yellowing in a “Valencia” sweet orange scion grafted onto US-1283 (VL/US-1283, incompatible).

The incompatibility symptoms in the US-1283 rootstock grafted with either “Bearss” or “Valencia” manifested as fluting or grooving of the rootstock stem and necrosis beneath the rootstock bark (**Figures 1**; **Supplementary Figure S1**). Stem grooving did not manifest until 2–3 months after grafting but became more noticeable during later stages of the experiment. Symptoms were more prominent and appeared earlier in US-1283 plants grafted with “Bearss”. The scion exhibited few symptoms except for leaf chlorosis, which was temporary and occurred in only a few plants 3–4 months after grafting. Some of the “Bearss”/US-1283 grafted trees also exhibited swelling of the scion directly above the graft union, which was more rarely observed in “Valencia”/US-1283 trees. Bud swelling, when present, was not evident until 3–4 months after grafting. Exudation of sap was observed in some “Bearss” plants on the graft union and from the stem bark directly above the graft union but did not occur until 9–10 months after grafting. An analysis of the grafted plant tissues against the Florida Division of Plant Industry (DPI)-mandated panel of pathogens (conducted by the DPI laboratory) yielded negative results, indicating that the symptoms and response observed were not caused by any tested infectious disease, including some viruses responsible for similar incompatibility symptoms in citrus, such as CTV and *Citrus tatter leaf virus* (CTLV, synonym *Apple stem grooving virus*, ASGV). In contrast, the same two scions grafted onto US-812 produced normal graft unions with no visible malformation of the rootstock, scion swelling, or chlorosis (**Figures 1**; **Supplementary Figure S1**).

### 3.2 RNA-seq analysis of differentially expressed genes

Gene expression profiling was used to compare the incompatible reactions of US-1283 grafted with “Bearss” (BL/US-1283) and “Valencia” (VL/US-1283) with US-812 compatible graft reactions (BL/US-812 and VL/US-812, respectively) as controls in the stem region above (AGU) and below (BGU) the graft unions. Four comparisons were made: (1) BL/US-1283 vs. BL/US-812 AGU, (2) BL/US-1283 vs. BL/US-812 BGU, (3) VL/US-1283 vs. VL/US-812 AGU, and (4) VL/US-1283 vs. VL/US-812 BGU (**Supplementary Tables S2**–**S5**). The differential comparisons are expressed as incompatible relative to compatible reactions and, for simplicity, are referred to as BL/US-1283 AGU, VL/US-1283 AGU, BL/US-1283 BGU, and VL/US-1283 BGU, respectively (see **Table 1**). The plants used for RNA extraction were uniform in their symptom expression, i.e., grooving and necrotic spots in the US-1283 rootstock and minimal to no swelling of the scion AGU (**Supplementary Figure S1**). US-1283 and US-812 have similar genetic backgrounds as they are both crosses of *C. reticulata* × *P. trifoliata*, although with different parental cultivars. Twenty-four libraries were constructed and sequenced, with three biological replicates for each of the four graft combinations and two regions relative to the graft union (**Supplementary Table S6**). In total, over 1.47 billion clean reads (adaptors and low-quality data removed) were obtained by Illumina sequencing, each library comprising more than 50 million reads. The average number of mapped transcripts was 20,778 per library, 88% of which was uniquely mapped and 3.68% was mapped to multiple loci. There are 24,533 protein-coding genes annotated in *C. clementina*.

**Table 1:** Summary of graft combinations analyzed using RNA-Seq and the number of differentially expressed genes (DEGs) detected in incompatible graft combinations compared to compatible combinations.

Short name	Comparison (incompatible vs. compatible)	Position relative to graft union	DEGs (*p*_Adj_ < 0.05)		
			Total	log2FC ≥1	log2FC ≤−1
BL/US-1283 AGU	BL/US-1283 - BL/US-812	AGU	102	33	37
VL/US-1283 AGU	VL/US-1283 - VL/US-812	AGU	205	78	46
BL/US-1283 BGU	BL/US-1283 - BL/US-812	BGU	4,749	1,446	1,159
VL/US-1283 BGU	VL/US-1283 - VL/US-812	BGU	2,680	1,053	649

DEGs, differentially expressed genes; log2FC, log2 of fold change.

BL, “Bearss” lemon, VL; “Valencia” sweet orange; AGU, above graft union; BGU, below graft union.

**Figure 2:**
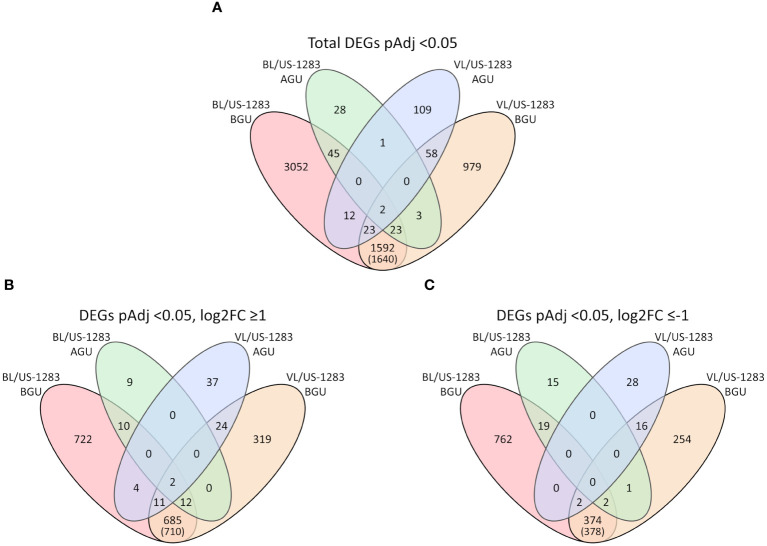
Venn diagrams show the numbers of differentially expressed genes (DEGs) above and below the graft union (AGU and BGU, respectively) in incompatible graft combinations with US-1283 relative to the compatible combinations with US-812 rootstock. **(A)** All DEGs (*p*_Adjusted_, *p*_Adj_ < 0.05) in the incompatible reactions. **(B)** DEGs induced in the incompatible reactions (*p*_Adj_ < 0.05 and log_2_ of fold change, log2FC ≥ 1). **(C)** DEGs repressed in the incompatible reactions (*p*_Adj_ < 0.05 and log2FC ≤ −1). Numbers in parenthesis indicate the total number of DEGs BGU common to both graft combinations, including those that were also DEGs AGU.

Pairwise comparisons, using the *C. clementina* annotation, between incompatible and compatible reactions revealed 102 and 205 significant DEGs (*p*_Adj_ < 0.05) in BL/US-1283 and VL/US-1283 AGU, respectively (**Table 1**). Of these, 33 and 78 DEGs were induced (*p*_Adj_ < 0.05, log2FC ≥1) and 37 and 46 were repressed (*p*_Adj_ < 0.05, log2FC ≤−1) in BL/US-1283 and VL/US-1283 AGU, respectively. In contrast, there were 4,749 and 2,680 significant DEGs in BL/US-1283 and VL/US-1283 BGU, of which 1,446 and 1,053 were induced and 1,159 and 649 were repressed, respectively. There were 1,640 common DEGs (710 induced and 378 repressed) between BL/US-1283 and VL/US-1283 BGU, and only 3 common DEGs between BL/US-1283 and VL/US-1283 AGU (**Figure 2**). Only two DEGs (both induced) were common to all graft combinations and tissue locations.

### 3.3 GO and KEGG pathway enrichment analysis and identification of key DEGs

**Figure 3:**
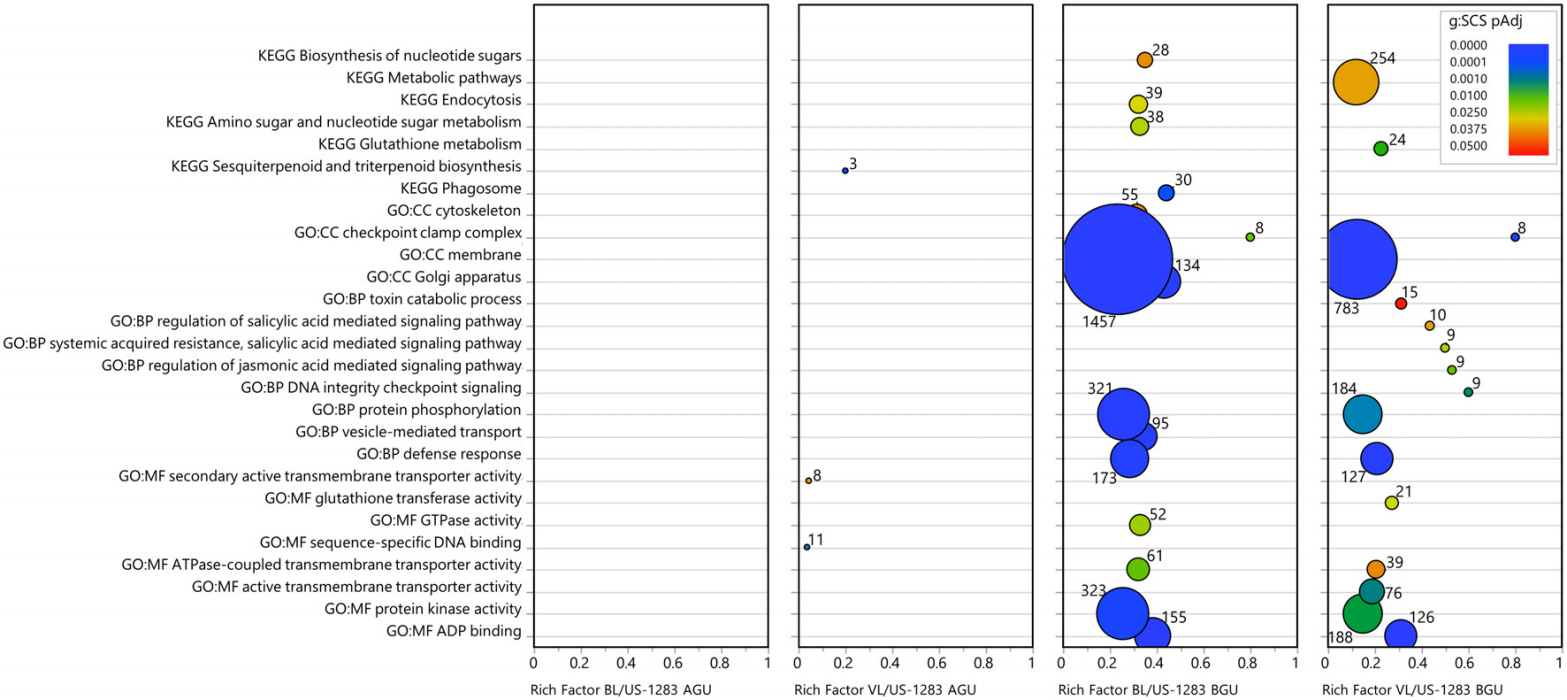
Categories of enriched GO and KEGG pathways of significant DEGs (*p*_Adj_ < 0.05). Rich factor is the number of DEGs in a category divided by the total number of genes in the category; GO, gene ontology; MF, molecular function; BP, biological process; CC, cellular components; KEGG, Kyoto Encyclopedia of Genes and Genomes pathway. Colors indicate the gene enrichment significance calculated by the g:SCS method (g:SCS *p*_Adj_); circle sizes are proportional to the number of DEGs in each category (indicated as numbers next to the circles).

**Figure 4:**
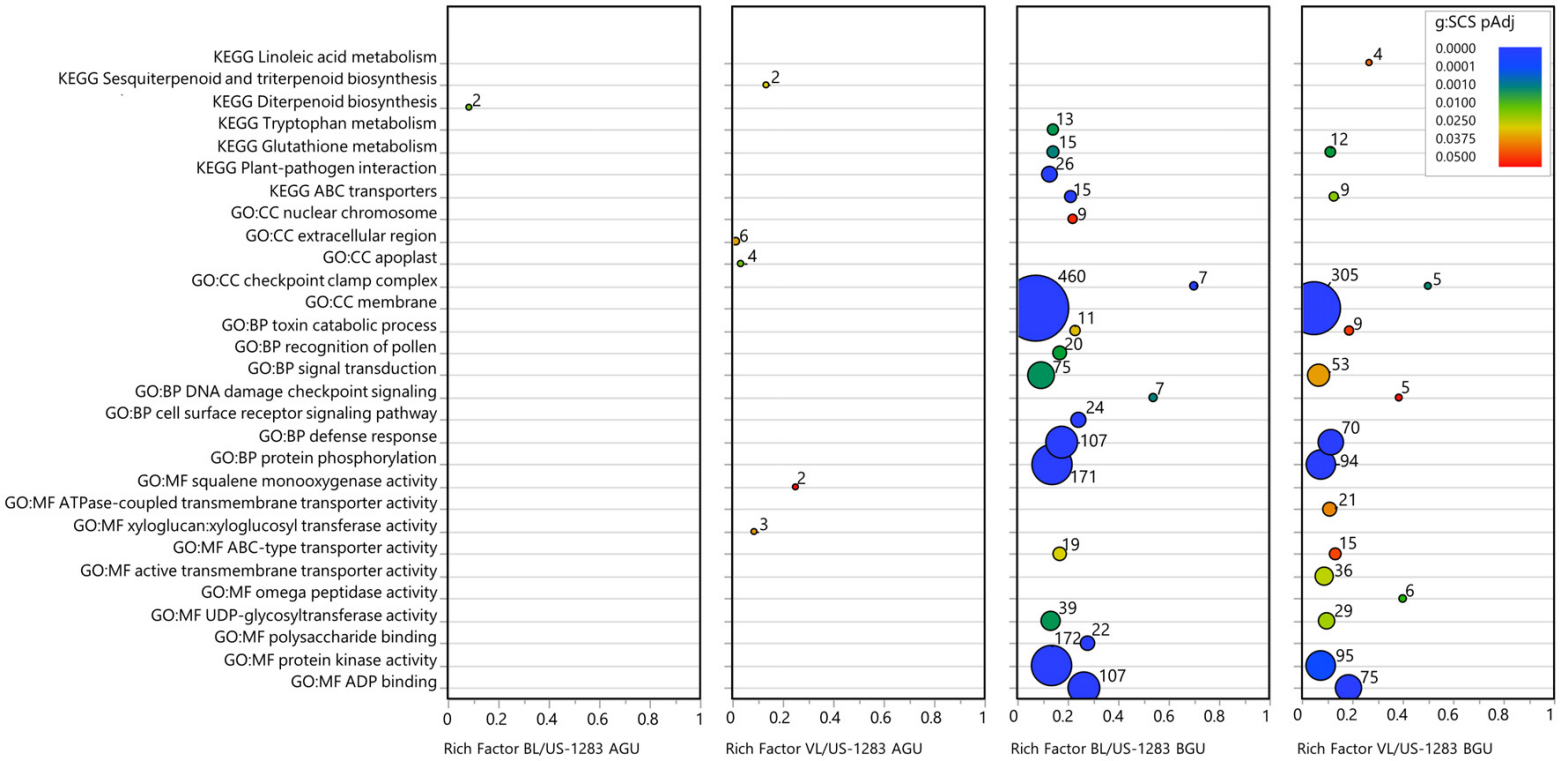
Categories of enriched GO and KEGG pathways of significantly induced DEGs [*p*_Adj_ < 0.05 and log_2_ Fold Change (log2FC) ≥1]. Rich factor is the number of DEGs in a category divided by the total number of genes in the category; GO, gene ontology; MF, molecular function; BP, biological process; CC, cellular components; KEGG, Kyoto Encyclopedia of Genes and Genomes pathway. Colors indicate the gene enrichment significance calculated by the g:SCS method (g:SCS *p*_Adj_); circle sizes are proportional to the number of DEGs in each category (indicated as numbers next to the circles).

**Figure 5:**
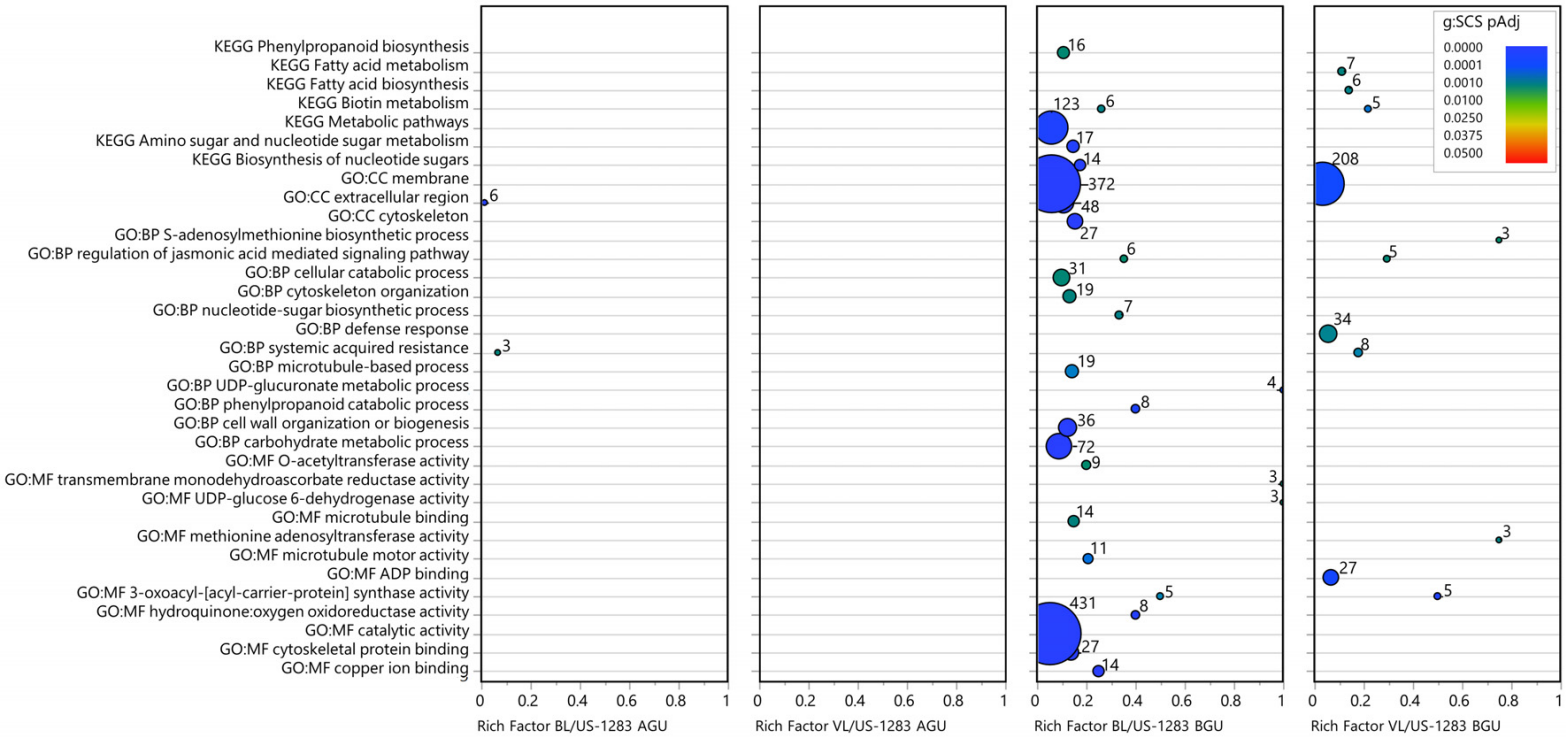
Categories of enriched GO and KEGG pathways of significantly repressed DEGs [*p*_Adj_ < 0.05 and log_2_ fold change (log2FC) ≤−1]. Rich factor is the number of DEGs in a category divided by the total number of genes in the category; GO, gene ontology; MF, molecular function; BP, biological process; CC, cellular components; KEGG, Kyoto Encyclopedia of Genes and Genomes pathway. Colors indicate the gene enrichment significance calculated by the g:SCS method (g:SCS *p*_Adj_); circle sizes are proportional to the number of DEGs in each category (indicated as numbers next to the circles).

Significant DEGs (*p*_Adj_ < 0.05) were subject to statistical enrichment analysis using both KEGG pathways and GO terms (**Figure 3**; **Supplementary Table S7**). The latter includes three categories: molecular function (MF), biological process (BP), and cellular components (CC). No terms were found to be significantly enriched in BL/US-1283 AGU, and only three terms were enriched in VL/US-1283 AGU. These were sequence-specific DNA binding (MF), secondary active transmembrane transporter activity (MF), and sesquiterpenoid and triterpenoid biosynthesis (KEGG). Terms significantly enriched in both BL/US-1283 BGU and VL/US-1283 BGU samples were ADP binding (MF), protein kinase activity (MF), ATPase-coupled transmembrane transporter activity (MF), defense response (BP), protein phosphorylation (BP), membrane (CC), and checkpoint clamp complex (CC). Enriched, significantly induced DEGs (*p*_Adj_ < 0.05, log2FC ≥1) (**Figure 4**; **Supplementary Table S7**) in BL/US-1283 AGU were associated with diterpenoid biosynthesis (KEGG). Significantly induced terms enriched in VL/US-1283 AGU were xyloglucan:xyloglucosyl transferase activity (MF), squalene monooxygenase activity (MF), apoplast (CC), extracellular region (CC), and sesquiterpenoid and triterpenoid biosynthesis (KEGG). ADP biding (MF), protein kinase activity (MF), UDP-glycosyltransferase activity (MF), ABC-type transporter activity (MF), protein phosphorylation (BP), defense response (BP), DNA damage checkpoint signaling (BP), signal transduction (BP), toxin catabolic process (BP), membrane (CC), checkpoint clamp complex (CC), ABC transporters (KEGG), and glutathione metabolism (KEGG) were induced terms enriched in both BL/US-1283 BGU and VL/US-1283 BGU. Enriched, significantly repressed DEGs (*p*_Adj_ < 0.05, log2FC ≤−1) (**Figure 5**; **Supplementary Table S7**) in BL/US-1283 AGU included the terms systemic acquired resistance (BP) and extracellular region (CC). No enriched terms were found in repressed DEGs of VL/US-1283 AGU. In BL/US-1283 BGU and VL/US-1283 BGU, commonly enriched terms in significantly repressed DEGs were 3-oxoacyl-[acyl-carrier-protein] synthase activity (MF), regulation of jasmonic acid (JA)-mediated signaling pathway (BP), membrane (CC), and biotin metabolism (KEGG).

Several of the enriched terms identified as common to the incompatible reactions BGU in US-1283 grafted with either scion were associated with stress response (DNA damage checkpoint signaling, checkpoint clamp complex, ABC transporters, glutathione metabolism, biotin metabolism, and toxin catabolic processes), including those associated with pathogen infection (defense response). In the defense response category, there were 173 DEGs in BL/US-1283 BGU and 127 genes in VL/US-1283 BGU (*p*_Adj_ < 0.05) (**Figure 3**; **Supplementary Table S8**), the majority of which were induced, 107 in BL/US-1283 BGU and 70 in VL/US-1283 BGU (**Figure 4**). Additionally, most of the annotations in this category, 118 in BL/US-1283 BGU and 93 in VL/US-1283 BGU (**Supplementary Table S8**), corresponded to putative disease resistance R genes. These proteins are part of a cellular surveillance system, acting as receptors involved in the detection of pathogens (Marone et al., 2013). Also in the defense response category were NONEXPRESSER OF PR GENES 1 (NPR1)-like genes 3 and 4 (NPR3 and NPR4, **Supplementary Table S8**), which are central in regulating the plant immune response to pathogens mediated by salicylic acid (SA) and JA (Fu et al., 2012; Ali et al., 2018; Ding et al., 2018). Accordingly, the NPR1-like genes appeared in other significantly enriched GO categories BGU [Regulation of JA-mediated signaling pathway, Systemic acquired resistance (SAR), SA-mediated signaling pathway, and Regulation of SA mediated signaling pathway] (**Figure 3**; **Supplementary Table S8**). Although gene enrichment showed three repressed DEGs in the SAR category in BL/US-1283 AGU (**Figure 5**), they were neither R genes nor NPR1-like. Two of the genes were annotated as Plant Natriuretic Peptide A (PNP-A), which are associated with photosynthesis, water homeostasis, and SA-mediated cell death (Lee et al., 2020). The third gene was annotated as bifunctional inhibitor/lipid-transfer protein/seed storage 2S albumin superfamily protein.

The Phenylpropanoid biosynthesis pathway (KEGG) was uniquely enriched in BL/US-1283 BGU and included 16 DEGs repressed with log2FC values ranging between −1 and −8 (**Figure 5**; **Supplementary Table S9**). The functional annotation of these DEGs identified eight transcripts encoding peroxidases catalyzing the oxidative polymerization of phenylpropanoids to guaiacyl, syringyl, and p-hydroxyphenol, the three units forming lignin (Gross, 1981; Yoon et al., 2015; Vanholme et al., 2019; Wang et al., 2022b). Accordingly, lignin metabolic process genes were repressed in BL/US-1283 BGU (*p*_Adj_ < 0.05, log2FC ≤−1) and were also significantly enriched (**Supplementary Tables S7**, **S9**), although they are not highlighted in **Figure 5**. Some of the same genes in these pathways were also repressed in VL/US-1283 BGU, despite this GO term not being significantly enriched in this graft combination (**Supplementary Table S9**). These genes are annotated as belonging to the laccase (LAC) gene family (**Supplementary Table S9**), which are involved in lignin production (Bai et al., 2023).

**Figure 6:**
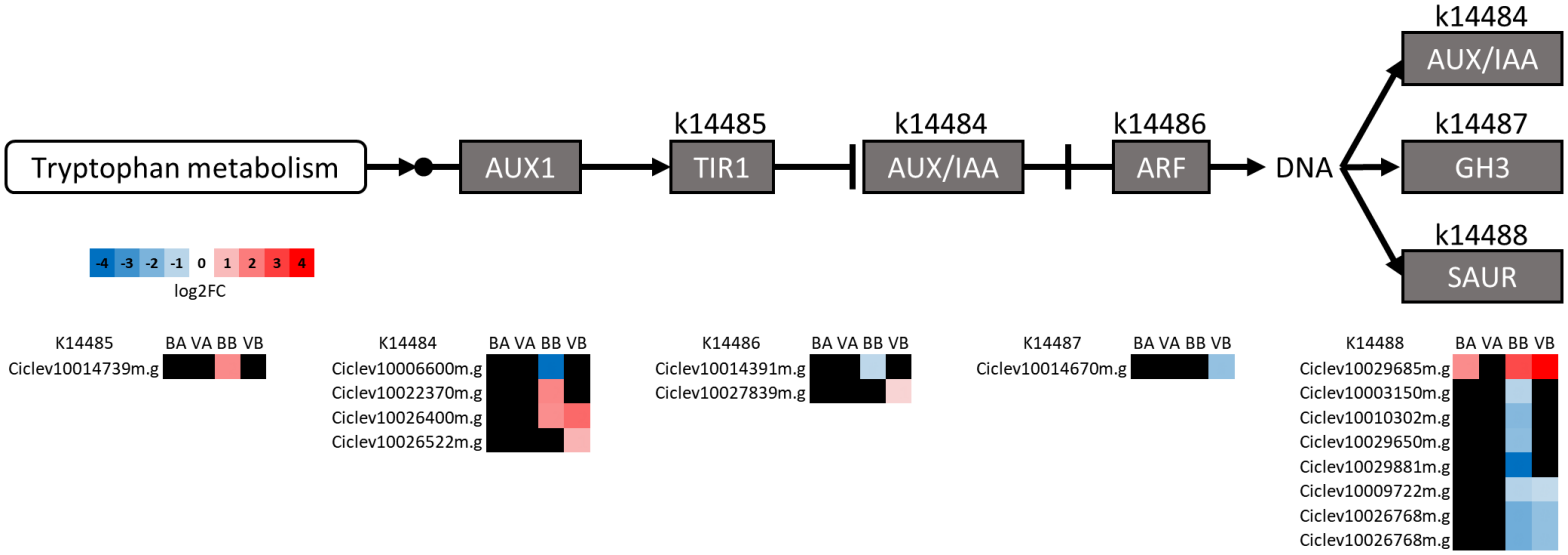
The Auxin signal transduction pathway reconstructed using the KEGG mapper reconstruction tool. KEGG ontology numbers (*k*) and the *C. clementina* gene IDs associated with them are indicated. Expression fold change (as log_2_ fold change, log2FC) for BL/US-1283 AGU (BA), VL/US-1283 AGU (VA), BL/US-1283 BGU (BB), and VL/US-1283 BGU (VB) is shown. Black squares are genes not significantly differentially expressed in the respective tissue. AUX 1, auxin influx carrier; TIR1, transport inhibitor response 1; AUX/IAA, auxin/indole-3-acetic acid responsive protein; ARF, auxin-responsive factor; GH3, Gretchen Hagen3 auxin-responsive family; SAUR, small auxin upregulated RNA family.

Auxins have been associated with graft formation and possibly play a role in incompatibility as well as cell enlargement and plant growth (Melnyk, 2017; Rasool et al., 2020; Zhai et al., 2021). Therefore, we examined those genes putatively involved in the auxin signal transduction pathway (**Figure 6**). The majority of DEGs in this pathway were found BGU. Of those, AUX/IAA genes were mostly induced, and small auxin upregulated RNA (SAUR) genes were mostly repressed. These proteins are involved in a variety of functions such as plant growth and development and stress response (Stortenbeker and Bemer, 2018; Zhang et al., 2021).

### 3.4 Validation of candidate genes

**Figure 7:**
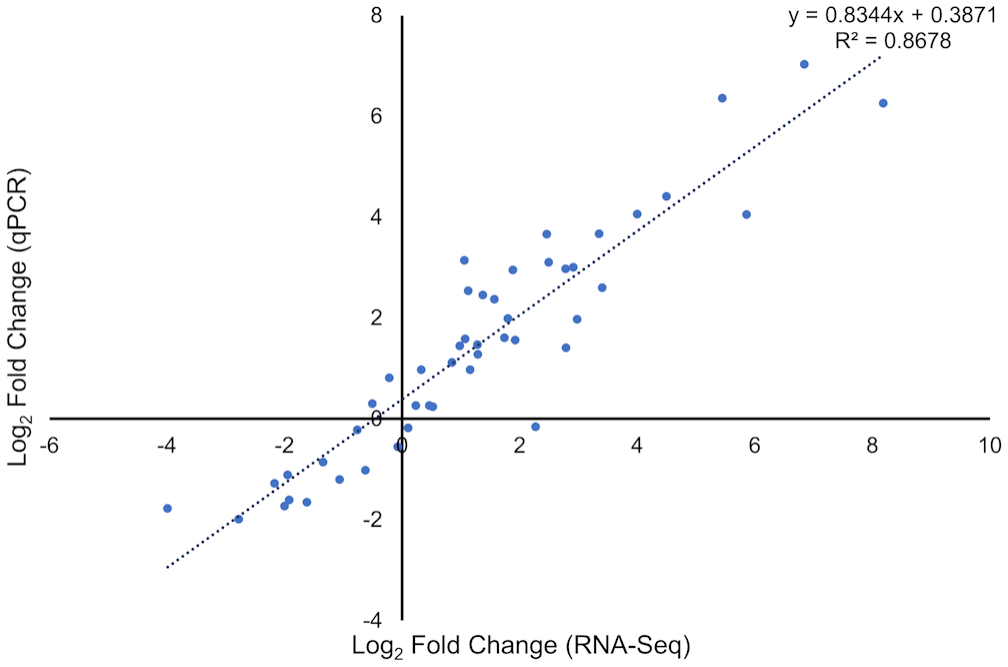
Correlation between the RNA-Seq expression data (*X* axis) and quantitative reverse transcription real-time PCR (qRT-PCR) expression (*Y* axis).

To verify the results of the RNA-Seq analysis, 11 DEGs with different expression patterns were selected for quantitative reverse transcription real-time PCR (qRT-PCR), using the tubulin gene as endogenous control (**Supplementary Table S1**). There was a strong positive correlation of gene expressions obtained with RNA-Seq and qRT-PCR (**Figure 7**; **Supplementary Figure S2**), confirming the transcriptome analysis.

## 4 Discussion

### 4.1 Incompatibility manifestation in US-1283

The grafting combinations produced for this experiment were part of a larger study to evaluate the compatibility of US-1283 with various scions. During these propagations, US-1283 was found to produce an incompatible interaction with “Bearss” lemon and other scions but not with “Hamlin” or “Valencia” sweet orange (Bowman and Albrecht, 2020). The present study intended to confirm these observations under controlled experimental conditions. Our experimental design included “Valencia”, which is the most propagated variety in Florida (Rosson, 2022), as a compatible control. However, contrary to previous results, in the propagations for this study, the VL/US-12183 combination developed the same symptoms as BL/US-1283, i.e., rootstock grooving and necrotic regions in the inner bark, albeit they were more moderate in intensity. US-1283 was demonstrated to have good genetic uniformity from seeds (Bisi et al., 2020), but there have been different graft compatibility reactions observed during various propagation events. Therefore, we compared the reactions of “Bearss” and “Valencia” on a single source of US-1283 with the same scions on US-812, a genetically similar rootstock that produced compatible grafts with these two scions. The incompatibility symptoms on US-1283 appeared a few months after grafting, and no pathogen infection was detected in any of the samples. Additionally, a previous study showed starch accumulation above the graft union, suggesting phloem degeneration and/or blockage at the graft union interface (Albrecht et al., 2021), which is generally associated with a translocated incompatibility (Mosse, 1962; Zarrouk et al., 2006). A molecular marker analysis performed after the completion of this study showed that the source trees used for deriving the seeds for this study had the same markers as the trees used for the original “Hamlin” propagations, confirming that the rootstock in our experimental plants was indeed US-1283 (K. Bowman, unpublished).

In both incompatible combinations (BL/US-1283 and VL/US-1283), the stem symptoms were more severe in the rootstock than the scion. This is reflected in the number of DEGs identified in the different tissues and graft combinations. Comparing BL/US-1283 with BL/US-812 (compatible), there were 102 DEGs AGU versus 4,749 DEGs BGU. Similarly, there were 205 DEGs AGU and 2,680 DEGs BGU when VL/US-1283 was compared to VL/US-812 (**Table 1**). This shows that considerably more transcriptional reprogramming occurred BGU than AGU.

Gene enrichment analysis of DEGs did not produce any categories of GO or KEGG pathway terms in BL/US-1283 AGU and only few terms in VL/US-1283 AGU (**Figure 3**). However, numerous transcriptional changes, including those associated with stress response, plant hormone signal transduction, and lignin precursor biosynthesis, were observed BGU in both incompatible combinations. The molecular responses to graft-induced stress were generally more intense when using BL as a scion, consistent with the severity of the symptoms observed. Graft incompatibility in citrus has been studied previously (He et al., 2018; He et al., 2022b). Incompatibility symptoms observed in these studies included yellowing and etiolation of the scion, whereas transcriptomic analysis revealed the disruption of carbohydrate metabolism and hormone signaling (auxin and abscisic acid) in the leaves resulting in the induction of stress response genes, similar to our observations.

### 4.2 Responses to oxidative stress and cell damage are induced by graft incompatibility

Genes in the glutathione metabolism were induced and enriched BGU in the US-1283 rootstock but not AGU in “Bearss” lemon or “Valencia” scions (**Figure 4**). This pathway is associated with the detoxification of reactive oxygen species (ROS) in the various cell compartments (Zechmann, 2020). Both biotic and abiotic stress triggers production of ROS (i.e., superoxide, hydrogen peroxide, etc.) in plants, which can cause oxidative damage to lipids, nucleic acids, and proteins as well as programmed cell death (Hasanuzzaman et al., 2019; Zechmann, 2020; Ye et al., 2021). ROS molecules also serve as signals regulating development during normal and stress conditions, triggering or modulating various signal transduction pathways (Hasanuzzaman et al., 2017; Mittler et al., 2022). Similarly, ABC (ATP binding cassette)-type transporter genes were induced BGU in the US-1283 rootstock but not AGU. This gene family has a variety of functions associated with transport across membranes such as import of nutrients, export of toxins, transport of ROS-related compounds, and transport of hormone signaling, including auxin and precursors (Do et al., 2021).

In the incompatible US-1283 rootstock, but not the “Bearss” lemon or “Valencia” scions, checkpoint clamp complex genes were also differentially expressed and significantly enriched (**Figure 3**). This complex is activated by DNA damage, leading to cell division arrest and DNA replication inhibition, DNA repair, and induction of apoptosis when DNA damage is severe (Kai, 2013; Liu, 2019; Zheng et al., 2022). Therefore, the bark necrosis and stem malformations observed in the rootstock could be, at least in part, the result of the reprogramming of this pathway. Genes involved in the biotin metabolism were significantly repressed in US-1283 rootstock in both incompatible graft combinations (BL/US-1283 and VL/US-1283, **Figure 5**). Biotin is a cofactor of enzymes involved in the biosynthesis and elongation of fatty acids (Alban et al., 2000). Accordingly, genes associated with fatty acid biosynthesis were also repressed.

### 4.3 Graft incompatibility symptoms resemble a pathogen-triggered defense response

Defense response genes were differentially expressed in the rootstocks of incompatible combinations. Of the DEGs in this GO category, 83 (48%) in BL/US-1283 BGU and 57 (44%) in VL/US-1283 BGU were induced (log2FC ≥ 1) putative R genes (**Supplementary Table S8**). Additionally, there was a set of NPR3 and NPR4 (NPR1-like) genes, key genes in the regulation of the SA- and JA-mediated signaling pathways and SAR (**Figures 3**, **5**; **Supplementary Table S8**) that were mostly repressed (log2FC ≤−1) in both BL/US-1283 BGU (six of nine DEGs) and VL/US-1283 BGU (five of nine DEGs). In Arabidopsis, only three of these genes have been identified (NPR1, 3, and 4), and NPR3/4 are receptors of SA as well as negative regulators (transcriptional corepressors) of SA-responsive genes and SAR (Zhang et al., 2006; Fu et al., 2012; Ding et al., 2018) and thus are essential regulators of plant immunity. NPR1 function is regulated transcriptionally and post-transcriptionally via the redox and polymerization/monomerization state of the protein and through interaction with other proteins, including NPR3/4 (Fu et al., 2012; Lee et al., 2015; Kumar et al., 2022). The NPR1-like genes of *Citrus* have not been fully functionally characterized; therefore, other functions or regulatory mechanisms for the more numerous paralogs in this species cannot be ruled out.

SAR is an important immune defense pathway in plants mediated by SA and associated with the accumulation of pathogenesis-related (PR) proteins at the point of pathogen entry and subsequently in distal parts of the plant. PR and SA biosynthesis-related genes were not among the significant DEGs identified in any of the tissues analyzed. Additionally, the differential expression of defense genes was observed only in the US-1283 rootstock and not the scion. Therefore, rather than a canonical SAR, a localized immune response seems to have been established, which used some of the same signaling pathways associated with the SA-mediated immune response. As suggested earlier, ROS, which is linked to early stages of plant defense against pathogens and programmed cell death, likely contributed to triggering and/or modulating this response as NPR1 activity is modulated post-transcriptionally via redox potential.

The plant material used in this study was obtained from disease-free, certified sources, and no evidence of infection with pathogens relevant to Florida was found. Although unlikely, the possibility that an untested pathogen was present cannot be completely dismissed. The symptoms observed in this study resemble in part those caused by the CTV and CTLV. CTV induces a variety of symptoms including stem pitting on grapefruit or sweet orange, seedling yellows in lemon and grapefruit, and the decline of many citrus species in graft combination with sour orange rootstock (Lee, 2015). CTLV especially causes bud union incompatibility manifested as fluting of the rootstock, necrosis at the bud union, and stunting or dwarfing in graft combinations with trifoliate orange (*P. trifoliata*) and trifoliate hybrid rootstocks (Miyakawa and Tsuji, 1988). However, symptoms are usually not apparent until three or more years (Roistacher, 1991). Neither pathogen was detected in our samples. Our results support the hypothesis that the stress associated with the incompatibility mimics a pathogen-induced defense response by causing the affected tissues BGU to be primed with the expression of R genes. As these genes function as plant immunity receptors, this is perhaps a mechanism that has evolved to reduce the risk of pathogen infection during stress conditions or when physical barriers intended to fend off pathogen attack have been compromised. This reaction was not transmitted systemically AGU, since no changes in defense genes were observed in the scions when compared to compatible graft unions on US-812.

### 4.4 Wood formation and secondary growth

The most visible incompatibility symptom in the rootstock were malformations in the stem. Wood formation is associated with the polymerization of lignin, from monomers biosynthesized from phenylpropanoids (Vanholme et al., 2019). Phenylpropanoids are a diverse class of natural compounds abundantly synthesized from phenylalanine during graft union formation (He et al., 2018; Amri et al., 2021; Hou et al., 2023). In addition to being precursors of lignin, they are also essential components of cell walls and play an important role as neutralizers of ROS and protectors against biotic and abiotic stress (Yoon et al., 2015; Deng and Lu, 2017). In this study, several genes involved in phenylpropanoid biosynthesis showed differential expression between BL/US-1283 and BL/US-812, particularly BGU. Eight of these DEGs, annotated as peroxidases catalyzing the oxidative polymerization of monolignols to form lignin, were all repressed in BL/US-1283 as compared to the compatible combination. Likewise, laccase genes, which in Arabidopsis regulate lignin polymerization (Bai et al., 2023), were also repressed. The repression of these two sets of genes possibly contributed to the inhibition of lignin biosynthesis and, together with apoptosis, to groove formation in the rootstock trunk below the graft union.

Auxin is an important regulator and modulator of plant growth, including vascular tissue formation, cell division and enlargement, and response to the environment (Luo et al., 2018; Tao and Estelle, 2018). Auxin is also involved in the formation of wood and secondary growth (Nilsson et al., 2008; Růžička et al., 2015; Smetana et al., 2019). Aux/IAA genes were differentially expressed in the US-1283 rootstock grafted with either BL or VL but not the corresponding scions. This was also the case for most of the SAUR genes differentially expressed. Aux/IAA proteins are co-receptors of auxin and are transcription repressors via interaction with ARF transcription factors. In Arabidopsis, members of the Aux/IAA family of proteins have redundant functions (Liscum and Reed, 2002; Tao and Estelle, 2018). Downstream of Aux/IAA are SAUR proteins, which are involved in cell elongation and senescence (Stortenbeker and Bemer, 2018; Zhang et al., 2021) as well as secondary growth and lignin biosynthesis (Oh et al., 2003; Wang et al., 2022a). Therefore, it is possible that repression of SAUR genes in the rootstock, but not the scion of incompatible reactions, also contributed to the observed stem malformations.

## 5 Conclusions

In grafted tree crops, new rootstocks are subjected to extensive studies of performance, including compatibility with some scions, prior to their release, but the evaluation of graft compatibility with all scions before release is rather impractical. Understanding the causes and mechanisms that lead to graft incompatibility in citrus is important to breeders and the industry and can potentially lead to more practical and rapid methods to identify possible graft incompatible combinations. Previous studies have characterized the graft incompatible response in citrus using transcriptome analysis (He et al., 2018; He et al., 2022a; He et al., 2022b); however, these studies concentrated on the response of the canopy, i.e., leaves. The present study evaluated the transcriptomic response of both grafting partners at the interface of the graft union. We found more genes differentially expressed in the rootstock, which was consistent with the incompatibility symptoms manifesting mostly below the graft union. Among the genes differentially expressed in the US-1283 rootstock, but not in either of the grafted scions, were those associated with ROS stress, phenylpropanoid/lignin biosynthesis, auxin-signal transduction, and defense. Similar graft incompatibility studies in other species have also revealed a transcriptional reprogramming of genes related to stress responses, ROS, plant defense, auxin signaling, and phenylpropanoid metabolism associated with heterografts and graft incompatible combinations in the graft union zone (Cookson et al., 2014; Chen et al., 2017); however, neither study differentiated between scion and rootstock tissues. Based on our results, we therefore hypothesize that US-1283 is recognizing and responding to the scion as non-self or is recognizing molecules from the scion as danger signals but not vice versa (Matzinger, 2002; Medzhitov and Janeway, 2002). The type of molecules from the scion that could be acting as danger-associated molecular patterns (DAMPs) in this case is unknown, but could be RNA, DNA, nucleotides, peptides, or other compounds (Ge et al., 2022). Alternatively, a signal from the scion, necessary for the formation and maintenance of a healthy graft union, may not be produced or perceived as such by the rootstock. We could not determine what was being produced by the scions that may be triggering the graft incompatibility. Other studies are underway to identify potential incompatibilities in other graft combinations. Identifying the signal molecule(s) inducing incompatibility would enable breeders to reduce the time needed to identify grafting problems. It may also make it possible to block these signals, or to breed rootstocks insensitive to the signals to promote the compatibility among new scion and rootstock selections. This study underscores the importance of studying both graft interacting partners to better understand the compatibility/incompatibility response. Furthermore, by advancing the understanding of the incompatible reaction in citrus, we identified potential specific gene expression targets for marker development.

## Data availability statement

The datasets presented in this study can be found in online repositories. The names of the repository/repositories and accession number(s) can be found in the article/**Supplementary Material**.

## Author contributions

VF: Conceptualization, Formal analysis, Investigation, Methodology, Validation, Visualization, Writing – original draft, Writing – review & editing. AF: Conceptualization, Formal analysis, Investigation, Methodology, Validation, Visualization, Writing – original draft, Writing – review & editing. BM: Investigation, Writing – review & editing. FY: Data curation, Formal analysis, Writing – review & editing. KB: Resources, Writing – review & editing. JC: Conceptualization, Funding acquisition, Resources, Writing – review & editing. UA: Conceptualization, Funding acquisition, Resources, Supervision, Writing – review & editing.
