# Supplementary figures and images for: Dissection of transcriptional events in graft incompatible reactions of “Bearss” lemon (Citrus limon) and “Valencia” sweet orange (C. sinensis) on a novel citrandarin (C. reticulata × Poncirus trifoliata) rootstock
Source: Front Plant Sci. 2024 Jun 20;15:1421734. doi: 10.3389/fpls.2024.1421734 (PMC11222572; doi:10.3389/fpls.2024.1421734)

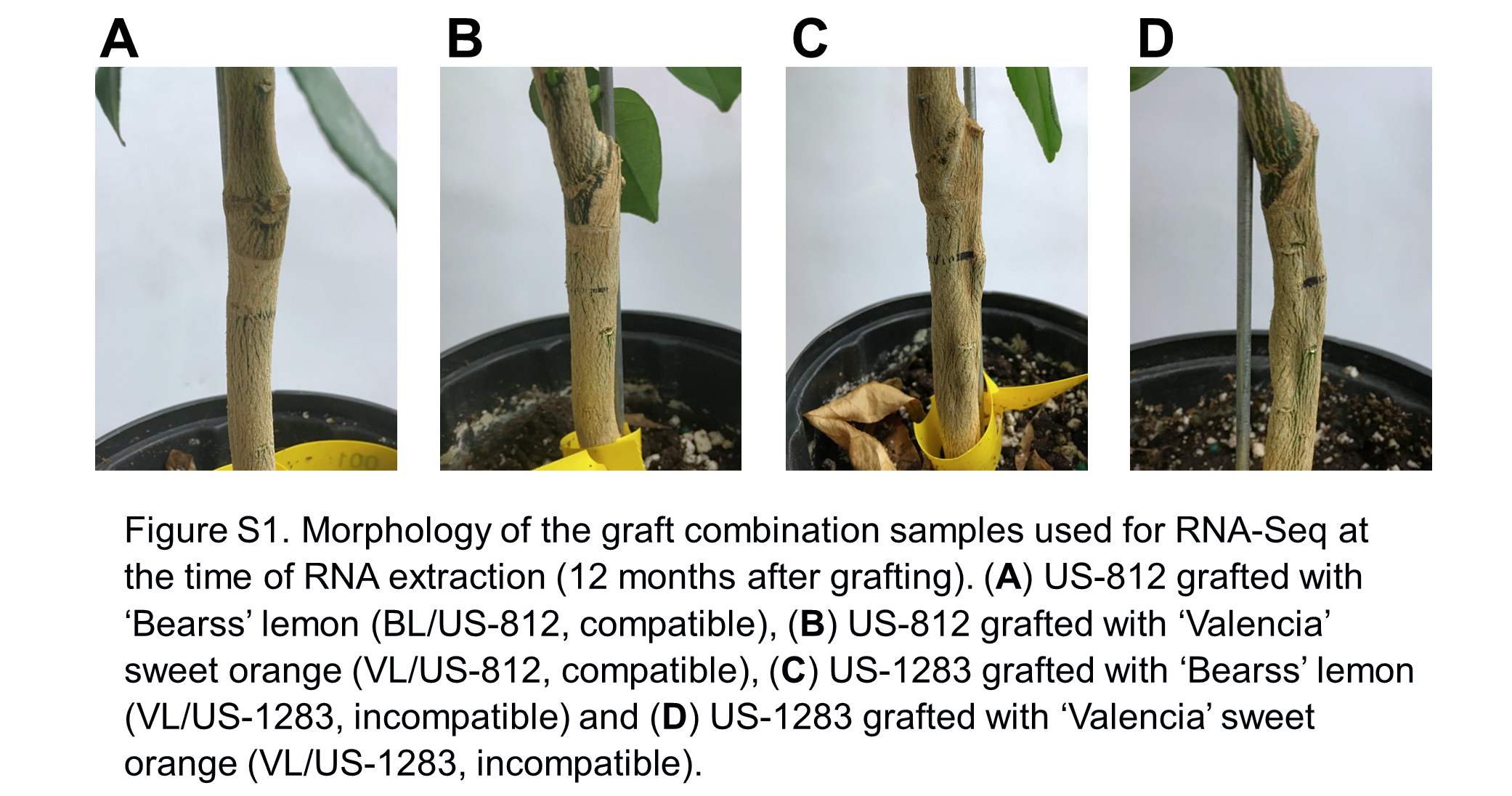

Supplement: Supplementary file 1 [file Image1.tif]

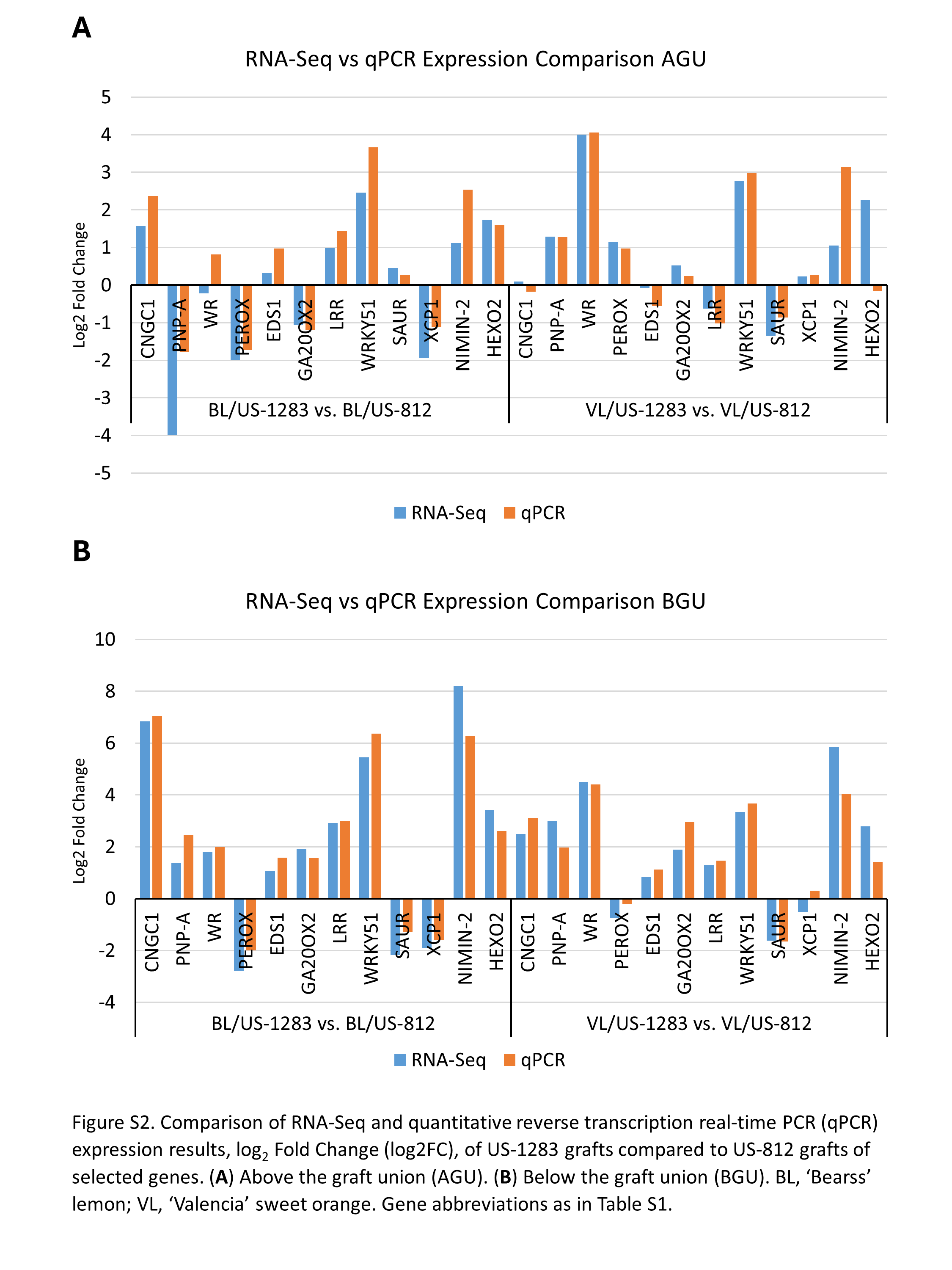

Supplement: Supplementary file 2 [file Image2.tif]
