# Supplementary material for: Dissection of transcriptional events in graft incompatible reactions of “Bearss” lemon (Citrus limon) and “Valencia” sweet orange (C. sinensis) on a novel citrandarin (C. reticulata × Poncirus trifoliata) rootstock
Source: Front Plant Sci. 2024 Jun 20;15:1421734. doi: 10.3389/fpls.2024.1421734 (PMC11222572; doi:10.3389/fpls.2024.1421734)
Supplement: Supplementary file 3 [file Table1.docx]

**Table S1.** List of primers used for the quantitative reverse transcription real-time PCR (qPCR) validation and their target genes.

| Target Gene | Function | Forward Primer | Reverse Primer |
| --- | --- | --- | --- |
| Ciclev10004563m.g (Cyclic nucleotide gated channel 1, CNGC1) | Defense response | CGACGGCAATGACCTTCTT | CCATGTAGCATCGGCAATCA |
| Ciclev10009878m.g (Plant natriuretic peptide A, PNP-A) | Plant hormone signal transduction | TTGCCATGATGGAGCAAGT | ATCAGCAATGACGGCGAA |
| Ciclev10010020m.g (Wound-responsive family protein, WR) | Wound Response | CATCTCTTCATCTCCAGCTTCC | AGCTCAAATCCATGACCCTTT |
| Ciclev10017786m.g (Peroxidase superfamily protein, PEROX) | Phenylpropanoid and lignin biosynthesis | GAAGGCTGTGATGGGTCTATTT | CAAGGCCTTTGCTTTCCTTATG |
| Ciclev10019035m.g (Alpha/beta-Hydrolases superfamily protein, EDS1) | Defense response | TTGGCCTGGTCCTGAATAAC | TTGTCCTGGTTTCTCCGTTT |
| Ciclev10020694m.g (Gibberellin 20 oxidase 2, GA20OX2) | Plant hormone signal transduction | CGGGATCATGGAGCTGTTAG | GCACGGTGGGTAGTAATTCA |
| Ciclev10007386m.g (Disease resistance family protein/LRR family protein, LRR) | Defense response | GCTCTCTGGCCTTTCTTTCT | GAGTGTGTTTGTTGCCATCAG |
| Ciclev10026733m.g (WRKY DNA-binding protein 51, WRKY51) | Defense response | GGAGGATGCCAAGTGAAGAA | GGCTCTCGTGATTGTGAGTT |
| Ciclev10026768m.g (SAUR-like auxin-responsive protein family, SAUR) | Plant hormone signal transduction | GTCCCGAAAGTTTGTGATTCC | CCATCCGTATTGTAACCGTAGA |
| Ciclev10028739m.g (Xylem cysteine peptidase 1, XCP1) | Vascular tissue differentiation | GGCTGCAATGGAGGTCTAAT | CACAAGTGCCTTCCTCCATAA |
| Ciclev10024601m.g (Beta-hexosaminidase 2, HEXO2) | Faty acid metabolism/defense response | GGTACAGGGTGATTGTGTCAT | ATCTCCGGAGGCTGATCATA |
| Ciclev10033073m.g (NIM1-interacting 2, NIMIN-2) | Defense response | TGCGATACTGAGGAGGATACA | TCGTCGAAATCTTCGGGTTC |
| Ciclev10015255m.g (Tubulin, TUB) | Endogenous control gene | ACATCCCGCCTAAGGGTCTG | TTCCTCCGAAACATAGCCGTA |
