# Supplementary material for: Dissection of transcriptional events in graft incompatible reactions of “Bearss” lemon (Citrus limon) and “Valencia” sweet orange (C. sinensis) on a novel citrandarin (C. reticulata × Poncirus trifoliata) rootstock
Source: Front Plant Sci. 2024 Jun 20;15:1421734. doi: 10.3389/fpls.2024.1421734 (PMC11222572; doi:10.3389/fpls.2024.1421734)
Supplement: Supplementary file 8 [file Table6.docx]

Table S6. RNA-Seq analysis library reads obtained against the *Citrus clementina* (Phytozome genome 182) v1.0 genome.

|  |  |  |  |  |  |  |  |  |  |  |  |
| --- | --- | --- | --- | --- | --- | --- | --- | --- | --- | --- | --- |
| **Sample name** | **Rootstock** | **Scion** | **Location** | **Raw-reads** | **Clean reads** | **Unique mapped** | **% Uniquely mapped** | **% Mapped multiple loci** | **Mapped transcripts** | **Total splices** | **% Unmapped** |
| **22A_S1** | US-1283 | BL | AGU | 54,347,501 | 54,331,730 | 47,581,021 | 87.58% | 4.55% | 20,952 | 42,822,542 | 7.69% |
| **10A_S2** | US-1283 | BL | AGU | 50,609,687 | 50,598,298 | 44,639,131 | 88.22% | 4.74% | 20,359 | 41,714,912 | 6.87% |
| **35A_S3** | US-1283 | BL | AGU | 63,698,678 | 63,686,139 | 56,420,318 | 88.59% | 4.24% | 20,756 | 52,120,526 | 6.99% |
| **29A_S4** | US-1283 | VL | AGU | 61,424,254 | 61,410,663 | 55,981,894 | 91.16% | 3.03% | 20,953 | 51,746,351 | 5.69% |
| **4A_S5** | US-1283 | VL | AGU | 62,678,275 | 62,663,076 | 56,871,601 | 90.76% | 3.03% | 21,113 | 51,886,251 | 6.11% |
| **18A_S6** | US-1283 | VL | AGU | 57,875,017 | 57,862,380 | 51,844,632 | 89.60% | 3.32% | 20,921 | 47,186,130 | 6.94% |
| **52A_S7** | US-812 | BL | AGU | 67,851,293 | 67,831,786 | 60,285,290 | 88.87% | 4.39% | 20,962 | 55,512,604 | 6.59% |
| **64A_S8** | US-812 | BL | AGU | 72,462,257 | 72,448,627 | 63,086,638 | 87.08% | 4.13% | 21,041 | 56,988,661 | 8.63% |
| **71A_S9** | US-812 | BL | AGU | 54,226,842 | 54,210,529 | 46,729,215 | 86.20% | 3.86% | 20,773 | 42,476,732 | 9.79% |
| **59A_S10** | US-812 | VL | AGU | 57,776,304 | 57,762,321 | 51,382,966 | 88.96% | 2.85% | 20,879 | 47,724,017 | 8.07% |
| **83A_S11** | US-812 | VL | AGU | 50,619,020 | 50,602,913 | 44,411,200 | 87.76% | 3.36% | 20,457 | 40,733,455 | 8.72% |
| **55A_S12** | US-812 | VL | AGU | 60,545,981 | 60,532,871 | 52,944,416 | 87.46% | 3.38% | 20,826 | 48,886,122 | 8.96% |
| **22B_S13** | US-1283 | BL | BGU | 50,377,475 | 50,364,622 | 43,740,466 | 86.85% | 3.81% | 20,613 | 40,244,204 | 9.15% |
| **10B_S14** | US-1283 | BL | BGU | 63,714,611 | 63,692,354 | 53,427,822 | 83.88% | 3.56% | 20,975 | 49,178,515 | 12.38% |
| **35B_S15** | US-1283 | BL | BGU | 67,703,703 | 67,686,949 | 57,951,410 | 85.62% | 4.15% | 20,903 | 52,741,925 | 9.99% |
| **29B_S16** | US-1283 | VL | BGU | 72,898,738 | 72,884,101 | 63,676,101 | 87.37% | 3.53% | 21,073 | 59,997,200 | 8.94% |
| **4B_S17** | US-1283 | VL | BGU | 61,330,148 | 61,316,125 | 54,509,690 | 88.90% | 3.34% | 20,982 | 51,089,917 | 7.64% |
| **18B_S18** | US-1283 | VL | BGU | 74,997,396 | 74,977,542 | 66,409,350 | 88.57% | 3.62% | 20,924 | 61,425,267 | 7.68% |
| **52B_S19** | US-812 | BL | BGU | 63,304,899 | 63,289,036 | 55,905,986 | 88.33% | 3.57% | 20,700 | 51,672,075 | 7.93% |
| **64B_S20** | US-812 | BL | BGU | 64,825,911 | 64,813,226 | 57,628,018 | 88.91% | 3.12% | 20,441 | 53,352,585 | 7.82% |
| **71B_S21** | US-812 | BL | BGU | 57,678,413 | 57,663,380 | 50,698,485 | 87.92% | 3.20% | 20,470 | 46,270,997 | 8.75% |
| **59B_S22** | US-812 | VL | BGU | 62,280,368 | 62,264,014 | 54,737,555 | 87.91% | 3.44% | 20,654 | 49,858,932 | 8.47% |
| **83B_S23** | US-812 | VL | BGU | 58,966,600 | 58,919,702 | 51,216,753 | 86.93% | 3.86% | 20,223 | 47,119,815 | 8.97% |
| **55B_S24** | US-812 | VL | BGU | 67,511,927 | 67,494,174 | 58,537,242 | 86.73% | 4.19% | 20,731 | 54,228,685 | 8.79% |
|  |  |  |  |  |  |  |  |  |  |  |  |
| BL, 'Bearss' lemon; VL, 'Valencia' sweet orange; AGU, above the graft union; BGU, below the graft union | | | | | | | | |  |  |  |
