# Supplementary material for: Dissection of transcriptional events in graft incompatible reactions of “Bearss” lemon (Citrus limon) and “Valencia” sweet orange (C. sinensis) on a novel citrandarin (C. reticulata × Poncirus trifoliata) rootstock
Source: Front Plant Sci. 2024 Jun 20;15:1421734. doi: 10.3389/fpls.2024.1421734 (PMC11222572; doi:10.3389/fpls.2024.1421734)
Supplement: Supplementary file 10 [file Table8.docx]

Table S8. Defense response and related GO terms DEGs (pAdj <0.05) and their expression level (log_2_ of their Fold Change, log2FC) below the graft union (BGU) in incompatible reactions of ‘Bearss’ lemon (BL) and ‘Valencia’ sweet orange (VL) grafted onto US-1283 compared to compatible US-812.

| **GO term** | | | | **log2FC BL/US-1283 BGU** | **log2FC VL/US-1283 BGU** | **Description** | **Type of protein** |
| --- | --- | --- | --- | --- | --- | --- | --- |
| DR |  |  |  | 8.970593107 | 9.510803053 | Ciclev10006105m.g MLP423 (MLP-like protein 423) |  |
| DR |  |  |  | 8.186505245 |  | Ciclev10033073m.g NIMIN-2 (NIM1-interacting 2) |  |
| DR |  |  |  | 7.837682233 | 8.285130573 | Ciclev10024838m.g (Disease resistance protein (CC-NBS-LRR class) family) | R |
| DR |  |  |  | 7.620814107 | 7.268244532 | Ciclev10024837m.g (Disease resistance protein (CC-NBS-LRR class) family) | R |
| DR |  |  |  | 7.415587647 | 7.81586062 | Ciclev10024821m.g (LRR and NB-ARC domains-containing disease resistance protein) | R |
| DR |  |  |  | 7.357498369 |  | Ciclev10024551m.g (LRR and NB-ARC domains-containing disease resistance protein) | R |
| DR |  |  |  | 7.168967624 | 5.305175488 | Ciclev10024583m.g (disease resistance protein (TIR-NBS-LRR class), putative) | R |
| DR |  |  |  | 7.122899749 | 6.341761712 | Ciclev10027428m.g (Disease resistance protein (CC-NBS-LRR class) family) | R |
| DR |  |  |  | 6.942592387 | 5.805115642 | Ciclev10024820m.g (NB-ARC domain-containing disease resistance protein) | R |
| DR |  |  |  | 6.723013864 | 9.672426347 | Ciclev10024504m.g (disease resistance protein (TIR-NBS-LRR class), putative) | R |
| DR |  |  |  | 6.567341593 | 9.038394203 | Ciclev10018550m.g (disease resistance protein (TIR-NBS-LRR class), putative) | R |
| DR |  |  |  | 6.174030295 | 6.438662798 | Ciclev10003446m.g MLO12 (Seven transmembrane MLO family protein) |  |
| DR |  |  |  | 6.155950377 | 5.25819452 | Ciclev10023686m.g (disease resistance protein (TIR-NBS-LRR class)) | R |
| DR |  |  |  | 5.756877819 | 4.335712899 | Ciclev10024832m.g (Disease resistance protein (CC-NBS-LRR class) family) | R |
| DR |  |  |  | 5.634635642 | 5.62317103 | Ciclev10027426m.g (Disease resistance protein (CC-NBS-LRR class) family) | R |
| DR |  |  |  | 5.231909686 |  | Ciclev10024119m.g (LRR and NB-ARC domains-containing disease resistance protein) | R |
| DR |  |  |  | 5.051966323 | 5.393518929 | Ciclev10027464m.g (Disease resistance protein (CC-NBS-LRR class) family) | R |
| DR |  |  |  | 4.840679068 | 4.66558051 | Ciclev10024814m.g (NB-ARC domain-containing disease resistance protein) | R |
| DR |  |  |  | 4.549994432 | 3.188943022 | Ciclev10027180m.g (Disease resistance protein (CC-NBS-LRR class) family) | R |
| DR |  |  |  | 4.527925644 |  | Ciclev10023584m.g (disease resistance protein (TIR-NBS-LRR class), putative) | R |
| DR |  |  |  | 4.447405663 |  | Ciclev10019599m.g (Disease resistance protein (TIR-NBS-LRR class) family) | R |
| DR |  |  |  | 4.40002749 | 4.309244758 | Ciclev10027069m.g RPS5 (Disease resistance protein (CC-NBS-LRR class) family) | R |
| DR |  |  |  | 4.341677678 |  | Ciclev10024379m.g (disease resistance protein (TIR-NBS-LRR class), putative) | R |
| DR |  |  |  | 4.285438283 | 2.181782089 | Ciclev10024480m.g (disease resistance protein (TIR-NBS-LRR class), putative) | R |
| DR |  |  |  | 4.233644727 |  | Ciclev10027525m.g (LRR and NB-ARC domains-containing disease resistance protein) | R |
| DR |  |  |  | 4.153023098 | 3.785394203 | Ciclev10027362m.g (Disease resistance protein (CC-NBS-LRR class) family) | R |
| DR |  |  |  | 4.041406106 |  | Ciclev10013431m.g (LRR and NB-ARC domains-containing disease resistance protein) | R |
| DR |  |  |  | 3.807258146 | 2.844564347 | Ciclev10027601m.g (NB-ARC domain-containing disease resistance protein) | R |
| DR |  |  |  | 3.777095711 |  | Ciclev10027169m.g (Disease resistance protein (CC-NBS-LRR class) family) | R |
| DR |  |  |  | 3.604024479 | 3.46470836 | Ciclev10018507m.g (LRR and NB-ARC domains-containing disease resistance protein) | R |
| DR |  |  |  | 3.595436183 | 3.102524664 | Ciclev10027535m.g (Disease resistance protein (CC-NBS-LRR class) family) | R |
| DR |  |  |  | 3.533583438 | 3.439281644 | Ciclev10001029m.g (alpha/beta-Hydrolases superfamily protein) |  |
| DR |  |  |  | 3.520203599 | 3.555150303 | Ciclev10013538m.g |  |
| DR |  |  |  | 3.509182016 |  | Ciclev10033078m.g (Bifunctional inhibitor/lipid-transfer protein/seed storage 2S albumin superfamily protein) |  |
| DR |  |  |  | 3.508570756 | 2.180246474 | Ciclev10018897m.g (Disease resistance protein (CC-NBS-LRR class) family) | R |
| DR |  |  |  | 3.424536731 | 6.185713941 | Ciclev10007011m.g (Disease resistance protein (TIR-NBS-LRR class) family) | R |
| DR |  |  |  | 3.324418521 | 2.607644261 | Ciclev10024297m.g (disease resistance protein (TIR-NBS-LRR class), putative) | R |
| DR |  |  |  | 3.286573797 |  | Ciclev10027596m.g (Disease resistance protein (CC-NBS-LRR class) family) | R |
| DR |  |  |  | 3.200289389 | 3.179639856 | Ciclev10024833m.g (Disease resistance protein (CC-NBS-LRR class) family) | R |
| DR |  |  |  | 3.130953794 | 2.684649519 | Ciclev10024835m.g (Disease resistance protein (CC-NBS-LRR class) family) | R |
| DR |  |  |  | 3.097209113 | 7.628049739 | Ciclev10024859m.g (Disease resistance protein (CC-NBS-LRR class) family) | R |
| DR |  |  |  | 3.090957433 | 3.290033996 | Ciclev10030085m.g PR4 (pathogenesis-related 4) |  |
| DR |  |  |  | 3.069794371 |  | Ciclev10017678m.g CERK1, LYSM RLK1 (chitin elicitor receptor kinase 1) |  |
| DR |  |  |  | 2.977201309 |  | Ciclev10024604m.g (disease resistance protein (TIR-NBS-LRR class), putative) | R |
| DR |  |  |  | 2.921459468 | 4.14782368 | Ciclev10024190m.g (disease resistance protein (TIR-NBS-LRR class), putative) | R |
| DR |  |  |  | 2.837317022 | 2.880441238 | Ciclev10024834m.g (Disease resistance protein (CC-NBS-LRR class) family) | R |
| DR |  |  |  | 2.782177576 | 2.825916163 | Ciclev10018540m.g (NB-ARC domain-containing disease resistance protein) | R |
| DR |  |  |  | 2.774250564 |  | Ciclev10027967m.g LACS2, LRD2 (long-chain acyl-CoA synthetase 2) |  |
| DR |  |  |  | 2.706523829 | 2.77216487 | Ciclev10018544m.g (disease resistance protein (TIR-NBS-LRR class), putative) | R |
| DR |  |  |  | 2.697195813 | 3.032339816 | Ciclev10018190m.g (NB-ARC domain-containing disease resistance protein) | R |
| DR |  |  |  | 2.586781686 |  | Ciclev10018594m.g (LRR and NB-ARC domains-containing disease resistance protein) | R |
| DR |  |  |  | 2.56973283 |  | Ciclev10022863m.g |  |
| DR |  |  |  | 2.569680524 | 1.787025904 | Ciclev10025693m.g (Disease resistance protein (CC-NBS-LRR class) family) | R |
| DR |  |  |  | 2.563589426 |  | Ciclev10006074m.g (Polyketide cyclase/dehydrase and lipid transport superfamily protein) |  |
| DR |  |  |  | 2.556003463 | 2.607233268 | Ciclev10025625m.g RPS5 (Disease resistance protein (CC-NBS-LRR class) family) | R |
| DR |  |  |  | 2.545682707 |  | Ciclev10024882m.g (LRR and NB-ARC domains-containing disease resistance protein) | R |
| DR |  |  |  | 2.534852984 | 2.541676029 | Ciclev10000326m.g (alpha/beta-Hydrolases superfamily protein) |  |
| DR |  |  |  | 2.507337267 | 2.563392503 | Ciclev10030075m.g RPS5 (Disease resistance protein (CC-NBS-LRR class) family) | R |
| DR |  |  |  | 2.489606631 | 1.551324182 | Ciclev10004042m.g (NB-ARC domain-containing disease resistance protein) | R |
| DR |  |  |  | 2.469599783 |  | Ciclev10000629m.g EDS1 (alpha/beta-Hydrolases superfamily protein) |  |
| DR |  |  |  | 2.410173691 | 2.323964855 | Ciclev10017820m.g (NB-ARC domain-containing disease resistance protein) | R |
| DR |  |  |  | 2.392718616 |  | Ciclev10025732m.g RPS5 (Disease resistance protein (CC-NBS-LRR class) family) | R |
| DR |  |  |  | 2.384887114 |  | Ciclev10023641m.g (disease resistance protein (TIR-NBS-LRR class), putative) | R |
| DR |  |  |  | 2.353046361 | 2.492498705 | Ciclev10024850m.g (Disease resistance protein (CC-NBS-LRR class) family) | R |
| DR |  |  |  | 2.314566872 |  | Ciclev10018586m.g (disease resistance protein (TIR-NBS-LRR class), putative) | R |
| DR |  |  |  | 2.305667294 | 1.771599518 | Ciclev10030216m.g RPS5 (Disease resistance protein (CC-NBS-LRR class) family) | R |
| DR |  |  |  | 2.296033565 | 2.023140335 | Ciclev10029998m.g RFL1 (RPS5-like 1) | R |
| DR |  |  |  | 2.292190085 |  | Ciclev10027594m.g (Disease resistance protein (CC-NBS-LRR class) family) | R |
| DR |  |  |  | 2.290241455 |  | Ciclev10015213m.g LHT1 (lysine histidine transporter 1) |  |
| DR |  |  |  | 2.209143959 | 1.386245979 | Ciclev10024735m.g (disease resistance protein (TIR-NBS-LRR class), putative) | R |
| DR |  |  |  | 2.151967187 | 2.572653434 | Ciclev10023418m.g (disease resistance protein (TIR-NBS-LRR class), putative) | R |
| DR |  |  |  | 2.127165205 |  | Ciclev10027198m.g (Disease resistance protein (CC-NBS-LRR class) family) | R |
| DR |  |  |  | 2.109270774 | 1.073907771 | Ciclev10023891m.g (disease resistance protein (TIR-NBS-LRR class), putative) | R |
| DR |  |  |  | 2.017973907 |  | Ciclev10024482m.g (disease resistance protein (TIR-NBS-LRR class), putative) | R |
| DR |  |  |  | 1.99507993 | 1.699525938 | Ciclev10024865m.g (Disease resistance protein (CC-NBS-LRR class) family) | R |
| DR |  |  |  | 1.899199013 | 2.124449091 | Ciclev10004284m.g (NB-ARC domain-containing disease resistance protein) | R |
| DR |  |  |  | 1.89460248 | 1.610464259 | Ciclev10023613m.g (LRR and NB-ARC domains-containing disease resistance protein) | R |
| DR |  |  |  | 1.839971294 | 0.937401536 | Ciclev10007376m.g (NB-ARC domain-containing disease resistance protein) | R |
| DR |  |  |  | 1.790366045 | 0.944390402 | Ciclev10030529m.g (LRR and NB-ARC domains-containing disease resistance protein) | R |
| DR |  |  |  | 1.728098623 |  | Ciclev10027589m.g (Disease resistance protein (CC-NBS-LRR class) family) | R |
| DR |  |  |  | 1.704007534 |  | Ciclev10023975m.g (disease resistance protein (TIR-NBS-LRR class), putative) | R |
| DR |  |  |  | 1.694334155 | 1.11135625 | Ciclev10017039m.g MLP28 (MLP-like protein 28) |  |
| DR |  |  |  | 1.649905174 | 1.697769161 | Ciclev10031557m.g (P-loop containing nucleoside triphosphate hydrolases superfamily protein) |  |
| DR |  |  |  | 1.64079995 | 1.354020612 | Ciclev10024848m.g (LRR and NB-ARC domains-containing disease resistance protein) | R |
| DR | JA | SAR | SA | 1.607612122 | 1.498925251 | Ciclev10031749m.g NPR3 (NPR1-like protein 3) | NPR |
| DR |  |  |  | 1.578046036 | 2.138710331 | Ciclev10022449m.g BAL, BAP2 (BON association protein 2) |  |
| DR |  |  |  | 1.576411124 |  | Ciclev10003578m.g (Disease resistance protein (TIR-NBS-LRR class) family) | R |
| DR |  |  |  | 1.5557445 | 1.136272161 | Ciclev10018509m.g (LRR and NB-ARC domains-containing disease resistance protein) | R |
| DR |  |  |  | 1.545145972 | 1.768973184 | Ciclev10023938m.g (disease resistance protein (TIR-NBS-LRR class), putative) | R |
| DR |  |  |  | 1.534396529 |  | Ciclev10031130m.g MLO12 (Seven transmembrane MLO family protein) |  |
| DR |  |  |  | 1.512448062 | 1.198905216 | Ciclev10018492m.g (LRR and NB-ARC domains-containing disease resistance protein) | R |
| DR |  |  | SA | 1.492850754 | 1.576490188 | Ciclev10014601m.g (MAC/Perforin domain-containing protein) |  |
| DR |  |  |  | 1.47541987 | 1.109432636 | Ciclev10027347m.g (Disease resistance protein (CC-NBS-LRR class) family) | R |
| DR |  |  |  | 1.470412549 | 1.137942815 | Ciclev10012995m.g |  |
| DR |  |  |  | 1.46087892 | 1.301381392 | Ciclev10023438m.g (disease resistance protein (TIR-NBS-LRR class)) | R |
| DR |  |  |  | 1.446081414 | 0.931626674 | Ciclev10024845m.g (Disease resistance protein (CC-NBS-LRR class) family) | R |
| DR |  |  |  | 1.397535799 |  | Ciclev10023260m.g (LRR and NB-ARC domains-containing disease resistance protein) | R |
| DR |  |  |  | 1.348538421 | 1.186522373 | Ciclev10024485m.g (disease resistance protein (TIR-NBS-LRR class), putative) | R |
| DR |  |  |  | 1.285850923 |  | Ciclev10027774m.g RBOHD (respiratory burst oxidase homologue D) |  |
| DR |  |  |  | 1.1725603 |  | Ciclev10022226m.g (Late embryogenesis abundant (LEA) hydroxyproline-rich glycoprotein family) |  |
| DR |  |  |  | 1.170136328 |  | Ciclev10000029m.g (LRR and NB-ARC domains-containing disease resistance protein) | R |
| DR |  |  |  | 1.126614454 |  | Ciclev10018556m.g (NB-ARC domain-containing disease resistance protein) | R |
| DR |  |  |  | 1.090059247 | 0.790965015 | Ciclev10027582m.g (LRR and NB-ARC domains-containing disease resistance protein) | R |
| DR |  |  |  | 1.067966579 |  | Ciclev10019035m.g EDS1 (alpha/beta-Hydrolases superfamily protein) |  |
| DR |  |  |  | 1.043433517 |  | Ciclev10018531m.g (LRR and NB-ARC domains-containing disease resistance protein) | R |
| DR |  |  |  | 1.022171667 |  | Ciclev10027511m.g (LRR and NB-ARC domains-containing disease resistance protein) | R |
| DR |  |  |  | 1.006406099 | 1.001116368 | Ciclev10018552m.g (disease resistance protein (TIR-NBS-LRR class), putative) | R |
| DR |  |  |  | 0.995951639 |  | Ciclev10007294m.g (NB-ARC domain-containing disease resistance protein) | R |
| DR |  |  |  | 0.978494923 | 0.848087948 | Ciclev10024856m.g (Disease resistance protein (CC-NBS-LRR class) family) | R |
| DR |  |  |  | 0.949712468 | 0.668937718 | Ciclev10018572m.g (disease resistance protein (TIR-NBS-LRR class), putative) | R |
| DR |  |  |  | 0.941003366 |  | Ciclev10024478m.g (NB-ARC domain-containing disease resistance protein) | R |
| DR |  |  |  | 0.938924806 |  | Ciclev10024836m.g (Disease resistance protein (CC-NBS-LRR class) family) | R |
| DR |  |  |  | 0.935460304 |  | Ciclev10024454m.g (LRR and NB-ARC domains-containing disease resistance protein) | R |
| DR |  |  |  | 0.917751613 |  | Ciclev10022655m.g |  |
| DR |  |  |  | 0.863131611 | 0.911359477 | Ciclev10027513m.g (Disease resistance protein (CC-NBS-LRR class) family) | R |
| DR |  |  |  | 0.848011864 |  | Ciclev10018699m.g RPS2 (NB-ARC domain-containing disease resistance protein) | R |
| DR |  |  |  | 0.834478924 |  | Ciclev10023333m.g ZAR1 (HOPZ-ACTIVATED RESISTANCE 1) |  |
| DR |  |  |  | 0.807818637 |  | Ciclev10027298m.g (Disease resistance protein (CC-NBS-LRR class) family) | R |
| DR |  |  |  | 0.80395422 |  | Ciclev10024868m.g (Disease resistance protein (CC-NBS-LRR class) family) | R |
| DR |  |  |  | 0.78893995 |  | Ciclev10023392m.g ZAR1 (HOPZ-ACTIVATED RESISTANCE 1) |  |
| DR |  |  |  | 0.765070578 |  | Ciclev10006796m.g (LRR and NB-ARC domains-containing disease resistance protein) | R |
| DR |  |  |  | 0.734595199 |  | Ciclev10006645m.g (NAD(P)-binding Rossmann-fold superfamily protein) |  |
| DR |  |  |  | 0.699569817 |  | Ciclev10024857m.g (Disease resistance protein (CC-NBS-LRR class) family) | R |
| DR |  |  |  | 0.681363967 |  | Ciclev10025023m.g (Histidine kinase-, DNA gyrase B-, and HSP90-like ATPase family protein) |  |
| DR |  |  |  | 0.671483845 |  | Ciclev10014088m.g (NB-ARC domain-containing disease resistance protein) | R |
| DR |  |  |  | 0.671205486 |  | Ciclev10024408m.g (Disease resistance protein (TIR-NBS-LRR class)) | R |
| DR |  |  |  | 0.648620434 |  | Ciclev10023658m.g (disease resistance protein (TIR-NBS-LRR class), putative) | R |
| DR |  |  |  | 0.622706034 |  | Ciclev10023911m.g (NB-ARC domain-containing disease resistance protein) | R |
| DR |  |  |  | 0.556723997 |  | Ciclev10024907m.g (Disease resistance protein (CC-NBS-LRR class) family) | R |
| DR |  |  |  | 0.403323543 |  | Ciclev10015259m.g (Emsy N Terminus (ENT)/ plant Tudor-like domains-containing protein) |  |
| DR |  |  |  | -0.362491847 |  | Ciclev10030725m.g MOS7 (nuclear pore complex protein-related) |  |
| DR |  |  |  | -0.362985721 |  | Ciclev10007328m.g (NB-ARC domain-containing disease resistance protein) | R |
| DR |  |  |  | -0.412312103 |  | Ciclev10018557m.g SCD1 (stomatal cytokinesis defective / SCD1 protein (SCD1)) |  |
| DR |  |  |  | -0.42635584 |  | Ciclev10005943m.g EIF(ISO)4E, EIF4E2, eIFiso4E, LSP, LSP1 (Eukaryotic initiation factor 4E protein) |  |
| DR |  |  |  | -0.439405586 |  | Ciclev10018681m.g (NB-ARC domain-containing disease resistance protein) | R |
| DR |  |  |  | -0.448756815 |  | Ciclev10019003m.g EIN1, ETR, ETR1 (Signal transduction histidine kinase, hybrid-type, ethylene sensor) |  |
| DR |  |  |  | -0.488364805 |  | Ciclev10016357m.g CES1 (Alkaline phytoceramidase (aPHC)) |  |
| DR |  |  |  | -0.504798624 |  | Ciclev10021104m.g (hydroxyproline-rich glycoprotein family protein) |  |
| DR |  |  |  | -0.534657728 |  | Ciclev10004188m.g (NB-ARC domain-containing disease resistance protein) | R |
| DR |  |  |  | -0.570888418 |  | Ciclev10005598m.g (Calcium-dependent lipid-binding (CaLB domain) family protein) |  |
| DR | JA | SAR | SA | -0.748373661 | -0.662324821 | Ciclev10031258m.g NPR3 (NPR1-like protein 3) | NPR |
| DR |  |  |  | -0.789490484 |  | Ciclev10018595m.g (Disease resistance protein (TIR-NBS-LRR class) family) | R |
| DR |  |  |  | -0.82519992 | -0.904042431 | Ciclev10007315m.g (NB-ARC domain-containing disease resistance protein) | R |
| DR |  |  |  | -0.838060889 |  | Ciclev10019254m.g EXO70E2 (exocyst subunit exo70 family protein E2) |  |
| DR |  |  |  | -0.877031244 |  | Ciclev10025091m.g (protein kinase family protein / peptidoglycan-binding LysM domain-containing protein) |  |
| DR | JA | SAR | SA | -0.905874125 | -0.942366167 | Ciclev10030929m.g NPR3 (NPR1-like protein 3) | NPR |
| DR |  |  |  | -0.932622289 | -1.248326836 | Ciclev10018626m.g (NB-ARC domain-containing disease resistance protein) | R |
| DR |  |  |  | -1.012023486 | -1.924411856 | Ciclev10017070m.g MLP423 (MLP-like protein 423) |  |
| DR |  |  |  | -1.025111358 |  | Ciclev10025325m.g MLO4 (Seven transmembrane MLO family protein) |  |
| DR |  |  |  | -1.222807506 | -1.536563442 | Ciclev10024831m.g (LRR and NB-ARC domains-containing disease resistance protein) | R |
| DR |  |  |  | -1.33087116 | -0.778750897 | Ciclev10021815m.g (Calcium-dependent lipid-binding (CaLB domain) family protein) |  |
| DR |  |  |  | -1.40610516 |  | Ciclev10024534m.g (disease resistance protein (TIR-NBS-LRR class), putative) | R |
| DR |  |  |  | -1.489834977 |  | Ciclev10017274m.g (Bifunctional inhibitor/lipid-transfer protein/seed storage 2S albumin superfamily protein) |  |
| DR | JA | SAR | SA | -1.580076247 | -2.145033104 | Ciclev10031627m.g NPR3 (NPR1-like protein 3) | NPR |
| DR | JA | SAR | SA | -1.911673033 | -2.44226109 | Ciclev10031432m.g NPR3 (NPR1-like protein 3) | NPR |
| DR |  |  |  | -2.04704619 | -2.138141952 | Ciclev10030657m.g (Disease resistance protein (CC-NBS-LRR class) family) | R |
| DR |  |  |  | -2.048285262 |  | Ciclev10006102m.g MLP423 (MLP-like protein 423) |  |
| DR |  |  |  | -2.072631271 | -1.89165666 | Ciclev10023258m.g (Disease resistance protein (TIR-NBS-LRR class) family) | R |
| DR |  |  |  | -2.106412427 |  | Ciclev10006849m.g (Calcium-dependent lipid-binding (CaLB domain) family protein) |  |
| DR |  |  |  | -2.337461658 | -2.786767138 | Ciclev10018458m.g (Disease resistance protein (TIR-NBS-LRR class) family) | R |
| DR |  |  |  | -2.485437529 | -2.244251439 | Ciclev10024176m.g (Disease resistance protein (TIR-NBS-LRR class) family) | R |
| DR |  |  |  | -2.541546202 | -1.586105037 | Ciclev10029271m.g (Late embryogenesis abundant (LEA) hydroxyproline-rich glycoprotein family) |  |
| DR | JA | SAR | SA | -2.54791291 |  | Ciclev10031115m.g NPR4 (NPR1-like protein 4) | NPR |
| DR |  |  |  | -2.939142124 | -1.797471249 | Ciclev10003660m.g RFL1 (RPS5-like 1) | R |
| DR |  |  |  | -3.100071343 | -2.759484737 | Ciclev10027225m.g (Disease resistance protein (CC-NBS-LRR class) family) | R |
| DR |  |  |  | -3.260330519 | -4.103027673 | Ciclev10024854m.g RPS5 (Disease resistance protein (CC-NBS-LRR class) family) | R |
| DR |  |  |  | -3.63200892 | -4.323502249 | Ciclev10025828m.g (Disease resistance protein (CC-NBS-LRR class) family) | R |
| DR | JA | SAR | SA | -3.691051592 | -4.543658423 | Ciclev10033908m.g NPR4 (NPR1-like protein 4) | NPR |
| DR |  |  |  | -4.537453211 | -4.377279531 | Ciclev10010265m.g (NB-ARC domain-containing disease resistance protein) | R |
| DR | JA | SAR | SA | -4.933097307 | -10.30696729 | Ciclev10033090m.g NPR4 (NPR1-like protein 4) | NPR |
| DR |  |  |  | -5.992940933 | -6.072475928 | Ciclev10018835m.g (Disease resistance protein (TIR-NBS-LRR class) family) | R |
| DR | JA | SAR | SA | -6.436183923 | -6.761195741 | Ciclev10031310m.g NPR4 (NPR1-like protein 4) | NPR |
| DR |  |  |  | -7.655558467 |  | Ciclev10010463m.g DIR1 (Bifunctional inhibitor/lipid-transfer protein/seed storage 2S albumin superfamily protein) |  |
| DR |  |  |  |  | -6.285516576 | Ciclev10023733m.g (disease resistance protein (TIR-NBS-LRR class), putative) | R |
| DR |  |  |  |  | -2.563354351 | Ciclev10013135m.g (Bifunctional inhibitor/lipid-transfer protein/seed storage 2S albumin superfamily protein) |  |
| DR |  |  |  |  | -2.555590014 | Ciclev10024092m.g (disease resistance protein (TIR-NBS-LRR class), putative) | R |
| DR |  |  |  |  | -2.379990776 | Ciclev10017543m.g (Heavy metal transport/detoxification superfamily protein) |  |
| DR |  |  |  |  | -2.239127336 | Ciclev10027800m.g RPP13 (NB-ARC domain-containing disease resistance protein) | R |
| DR |  |  |  |  | -2.163005348 | Ciclev10003293m.g (Disease resistance protein (TIR-NBS-LRR class) family) | R |
| DR |  |  |  |  | -1.776252512 | Ciclev10013132m.g (Bifunctional inhibitor/lipid-transfer protein/seed storage 2S albumin superfamily protein) |  |
| DR |  |  |  |  | -1.711121879 | Ciclev10000339m.g SBT5.2 (Subtilisin-like serine endopeptidase family protein) |  |
| DR |  |  |  |  | -1.653091724 | Ciclev10033080m.g (Bifunctional inhibitor/lipid-transfer protein/seed storage 2S albumin superfamily protein) |  |
| DR |  |  |  |  | -1.359534111 | Ciclev10005095m.g WRKY27 (WRKY DNA-binding protein 27) |  |
| DR |  |  |  |  | -1.316280008 | Ciclev10017638m.g MLP28 (MLP-like protein 28) |  |
| DR |  |  |  |  | -1.305799013 | Ciclev10017324m.g MLP165 (MLP-like protein 165) |  |
| DR |  |  |  |  | -1.170618708 | Ciclev10023982m.g (disease resistance protein (TIR-NBS-LRR class), putative) | R |
| DR |  |  |  |  | -1.130966557 | Ciclev10010508m.g LEA14, LSR3 (Late embryogenesis abundant protein) |  |
| DR |  |  |  |  | -1.007371841 | Ciclev10029536m.g PR4 (pathogenesis-related 4) |  |
| DR | JA | SAR | SA |  | -0.973624159 | Ciclev10033569m.g NPR3 (NPR1-like protein 3) | NPR |
| DR |  |  |  |  | -0.947658164 | Ciclev10017794m.g RPP13 (NB-ARC domain-containing disease resistance protein) | R |
| DR |  |  |  |  | -0.523244915 | Ciclev10010250m.g (NB-ARC domain-containing disease resistance protein) | R |
| DR |  |  |  |  | 0.562676333 | Ciclev10027705m.g (NB-ARC domain-containing disease resistance protein) | R |
| DR |  |  |  |  | 0.579965658 | Ciclev10007304m.g (NB-ARC domain-containing disease resistance protein) | R |
| DR |  |  |  |  | 0.647277764 | Ciclev10023669m.g (NB-ARC domain-containing disease resistance protein) | R |
| DR |  |  |  |  | 0.710418573 | Ciclev10030540m.g (NB-ARC domain-containing disease resistance protein) | R |
| DR |  |  |  |  | 0.716360566 | Ciclev10024456m.g (disease resistance protein (TIR-NBS-LRR class), putative) | R |
| DR |  |  |  |  | 0.718255258 | Ciclev10024285m.g (disease resistance protein (TIR-NBS-LRR class), putative) | R |
| DR |  |  |  |  | 0.718926507 | Ciclev10024606m.g (Disease resistance protein (TIR-NBS-LRR class) family) | R |
| DR |  |  |  |  | 0.724848494 | Ciclev10010451m.g RPM1, RPS3 (NB-ARC domain-containing disease resistance protein) | R |
| DR |  |  |  |  | 0.806051417 | Ciclev10023391m.g (Disease resistance protein (TIR-NBS-LRR class) family) | R |
| DR |  |  |  |  | 1.040729004 | Ciclev10023653m.g (disease resistance protein (TIR-NBS-LRR class), putative) | R |
| DR |  |  |  |  | 1.986459001 | Ciclev10003985m.g (NB-ARC domain-containing disease resistance protein) | R |
| DR |  |  |  |  | 2.337395394 | Ciclev10007349m.g (NB-ARC domain-containing disease resistance protein) | R |
| DR |  |  |  |  | 2.681687531 | Ciclev10028092m.g MLO6 (Seven transmembrane MLO family protein) |  |
| DR |  |  |  |  | 5.574132689 | Ciclev10027214m.g (Disease resistance protein (CC-NBS-LRR class) family) | R |

**GO Term:** DR, Defense response; JA, Regulation of jasmonic acid mediated signaling pathway; SAR, Systemic acquired resistance, salicylic acid mediated signaling pathway; SA, Regulation of salicylic acid mediated signaling pathway.

**log2FC**, log_2_ Fold Change; **BL**, ‘Bearss’ lemon; **VL**, ‘Valencia’ sweet orange; **BGU**, below the graft union. Red, induced; Blue, repressed.

**Description:** *C. clementina* gene ID, gene abbreviation, and gene annotation (in parenthesis).

**Type of Protein:**  R, disease resistance; NPR, NONEXPRESSER OF PR GENES transcriptional activator (NPR1) or repressor (NPR3, NPR4).
